# Transplanted human photoreceptors differentially survive, incorporate, and mature in mildly and severely degenerated mouse retinae

**DOI:** 10.1016/j.omta.2026.201866

**Published:** 2026-10-14

**Authors:** Maria Pavlou, Karen Tessmer, Juliane Hammer, Thomas Kurth, Aikaterini Makri, Celine Palitza, Berta Coll-San Martin, Fabian Rost, Marius Ader

**Affiliations:** 1 Center for Regenerative Therapies Dresden (CRTD), Center for Molecular and Cellular Bioengineering (CMCB), Dresden University of Technology (TUD), 01307 Dresden, Germany; 2 Core Facility Electron Microscopy and Histology, Center for Molecular and Cellular Bioengineering (CMCB), Dresden University of Technology (TUD), 01307 Dresden, Germany; 3 Max Planck Institute of Molecular Cell Biology and Genetics, 01307 Dresden, Germany

**Keywords:** retinal degeneration, hiPSCs, retinal organoid, photoreceptor, transplantation, donor-host interactions, Müller glia, integration, disease severity

## Abstract

Photoreceptor transplantation is considered a disease-agnostic therapeutic strategy for retinal degenerative diseases with highly heterogeneous genetic, molecular, and cellular pathologies. While integration of human photoreceptors enriched from stem-cell-derived retinal organoids was noted in previous preclinical studies, the potential influence of retinal degeneration severity on transplantation efficiency has not been systematically assessed. Here, we employed mice presenting mild or severe retinal degeneration as recipients for human induced pluripotent stem cell-derived photoreceptors. Donor cells formed multicellular clusters that started to interact with Müller glia processes from 3 weeks post transplantation (wpt) in mildly degenerated retinae, resulting in structural integration and maturation, characterized by inner/outer segment and synapse formation by 26 wpt. In contrast, in severely degenerated hosts, donor photoreceptors remained mainly scattered and showed limited incorporation or signs of maturation. Differential maturation of donor cells in mild vs. severe hosts was confirmed by single-cell RNA-sequencing analysis. However, transplantation at the beginning of the degeneration process of the severe model allowed structural integration and maturation of donor photoreceptors despite complete loss of endogenous photoreceptors over time. The study thus shows that survival, integration, and maturation of donor photoreceptors depend on the degenerative retinal microenvironment shaping significantly the transplantation efficiency.

## Graphical abstract

**Figure:**
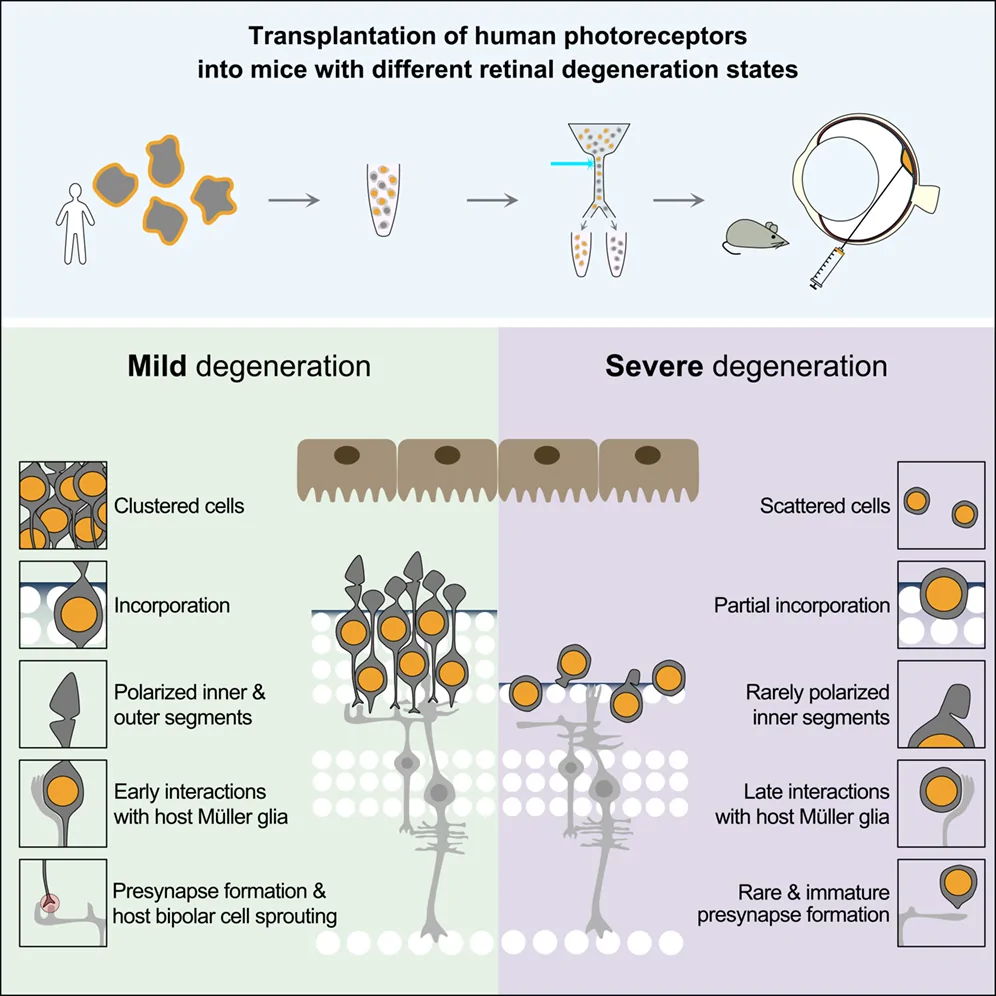

## Teaser

Human photoreceptors, isolated from iPSC-derived retinal organoids, survived, structurally integrated, and matured to a lesser extent in severe versus mild retinal degeneration mouse models, highlighting the influence of the host environment on transplantation efficiency.

## Introduction

Retinal degenerative diseases (RDDs), such as retinitis pigmentosa and age-related macular degeneration, culminate in loss of photoreceptors, the neuronal light-sensitive cells of the retina. As this cell type cannot regenerate in the mammalian retina, cell replacement is considered as a general disease-agnostic therapeutic strategy for vision restoration. Emphasis has so far been placed on the generation of candidate donor cells from embryonic stem cells (ESCs) or induced pluripotent stem cells (iPSC) in order to produce retinal organoids and isolate a transplantable population of photoreceptors. Indeed, previous studies provide evidence for incorporation of human ESC-/iPSC-derived photoreceptors into mouse models of retinal degeneration and restoration of some photoreceptor-mediated vision,^1^^,^^2^^,^^3^^,^^4^ culminating in the initiation of a recently started first clinical trial (NCT06789445, BlueRock Therapeutics, ClinicalTrials.gov).

RDD pathologies are characterized by high causative and phenotypic heterogeneity and thus a diverse extent of photoreceptor degeneration, accompanied by inner retinal remodeling. Recent transplantation studies used different RDD mouse models ranging from moderate to severe photoreceptor loss.^5^ While signs of donor photoreceptor incorporation including synapse formation, inner/outer segment generation, and interaction with Müller glia cells as well as some functional rescue were reported, signs of photoreceptor maturation and the degree of donor cell integration appeared highly varied in the different studies.^2^^,^^4^^,^^6^^,^^7^ Thus, there is currently limited knowledge as to what extent the degeneration status influences donor-host interaction and maturation of donor photoreceptors, parameters that are key for functional integration and vision restoration.

To systematically evaluate the influence of the degeneration status on photoreceptor transplants, we utilized in this study the cone-deficient mouse model Cone photoreceptor function loss 1 (Cpfl1)^8^ and the cone- and rod-deficient mouse model Cpfl1/Rhodopsin^−/−^ (tg(Cpfl1/Rho^−/−^)),^9^ representing mild and severe retinal degeneration, respectively. Donor photoreceptors were isolated from retinal organoids differentiated from a human photoreceptor-specific reporter iPSC line (CRX-mCherry-iPSC),^10^^,^^11^ followed by subretinal delivery into host mice.^2^^,^^12^ Histological analysis and single-cell gene-expression profiling revealed that transplanted photoreceptors can survive long term (up to 6 months) in both mildly and severely degenerated retinae, although they significantly differ in their distribution, integration, and differentiation status, with drastically improved cluster formation, donor-host interaction, and photoreceptor maturation within the mild degeneration environment of Cpfl1 mice. However, when transplanted at an early stage of retinal degeneration, cluster formation, structural integration, and maturation of donor cells was also observed in the severe tg(Cpfl1/Rho^−/−^) model, despite complete loss of endogenous photoreceptors over time.

## Results

### Validation of a human photoreceptor reporter iPSC line to produce a transplantable population of photoreceptors

A human iPSC line, bearing the mCherry tag under the control of a photoreceptor-specific cone-rod homeobox (CRX) promoter, was used to produce human retinal organoids (HROs).^10^ Day-200 (D200) HROs presented a laminated structure, resembling organization of the human retina, where Müller glia nuclei (SRY-box transcription factor 2 [SOX2]) resided beneath the photoreceptor nuclear layer (mCherry) (Figure S1A). Müller glia processes, indicated by staining against glial glutamate/aspartate transporter (GLAST/SLC1A3), extended over the photoreceptor nuclear layer, forming a notional outer limiting membrane (OLM). Inner- and outer-segment-like protrusions of photoreceptors were located directly above this structure, visible under differential interference contrast (DIC) microscopy (Figure S1A). A significant number of mCherry^+^ cells co-stained with the cone-specific marker arrestin 3 (ARR3) and signs of cone maturation included expression of opsins of short (OPN1SW) and medium/long (OPN1M/LW) wavelength, although labeling was not restricted to outer-segment-like protrusions but were found throughout the whole cell body (Figure S1B), indicative of an earlier state of differentiation.

To obtain an enriched fraction of transplantable photoreceptor cells, D200 HROs were dissociated into a single-cell suspension and underwent fluorescence-activated cell sorting (FACS) for mCherry^+^ cells, corresponding to ca. 65% of the alive (i.e., DAPI^−^) cell population (Figure S1C). Photoreceptor identity of sorted mCherry^+^ cells was validated by immunocytochemistry against the pan-photoreceptor human-specific marker recoverin (hRCVRN). Nevertheless, their characteristic photoreceptor architecture was lost, presenting a round morphology, likely attributed to shear stress during the steps of dissociation and FACS (Figure S1D).

### Characterization of degeneration status and Müller glia reactivity in hosts

Before delivery of donor cells into hosts, the mouse models were characterized to define the extent of photoreceptor loss and potential Müller glia reactivity. In this study, two mouse models presenting different degeneration states were used to assess how the severity of retinal degeneration of the host might have an influence on the survival, incorporation, and maturation of donor photoreceptors. Cpfl1 mice carry indel mutations in the phosphodiesterase 6C gene. They are characterized by dysfunctional cones and their subsequent loss, as indicated by staining against ARR3 (Figure 1A), but have remaining rod photoreceptors (rhodopsin [RHO]) and thus an outer nuclear layer (ONL) (Figure 1B), representing a case of mild retinal degeneration. In contrast, tg(Cpfl1/Rho^−/−^) mice, a strain resulting from crossing Cpfl1 to rhodopsin knockout mice, presented severe retinal degeneration with an almost complete loss of cones and rods and, therefore, also the ONL, by the age of 16 weeks (Figures 1A and 1B).

**Figure 1:**
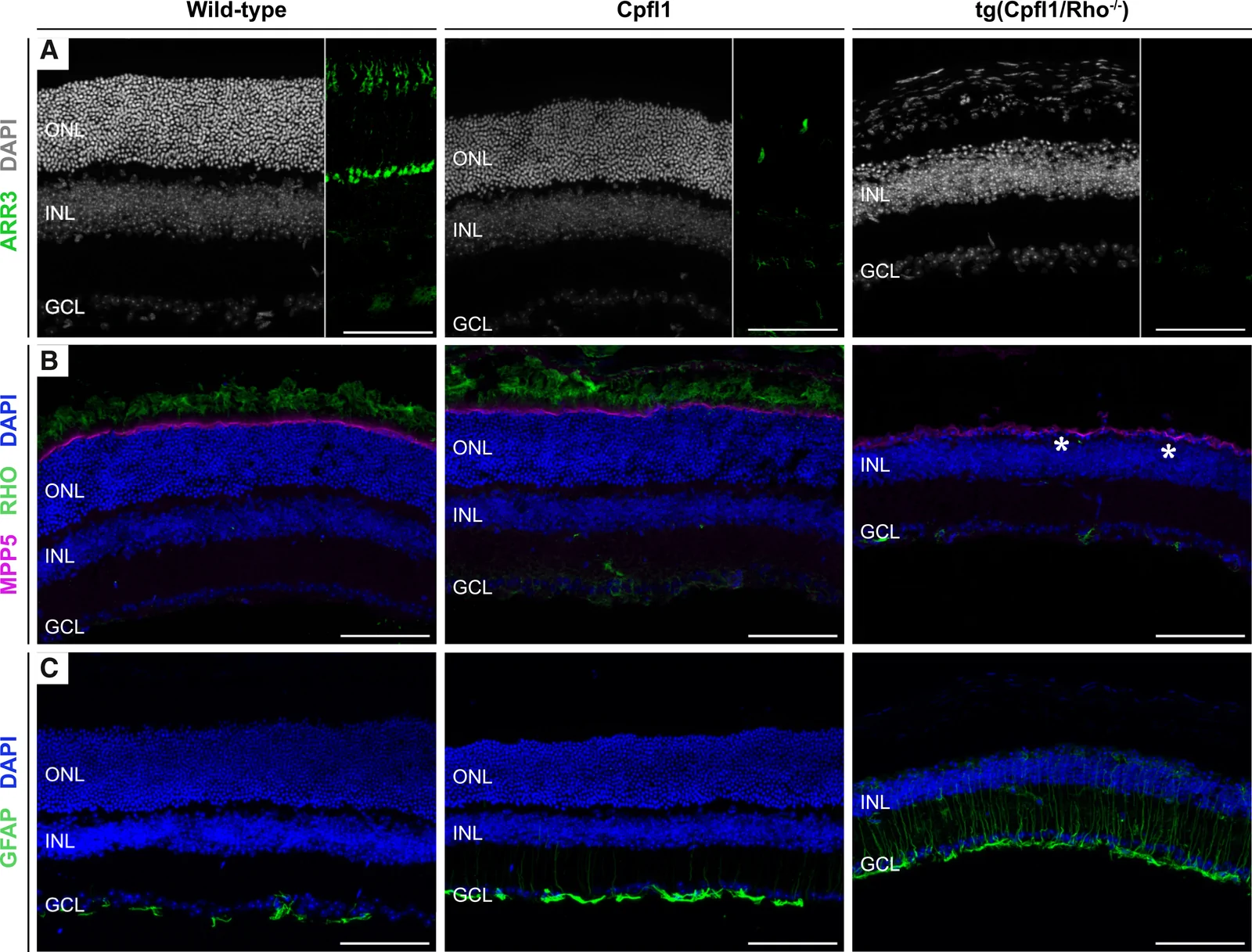
Cpfl1 and tg(Cpfl1/Rho^−/−^) mice present different states of photoreceptor degeneration and Müller glia reactivity (A) Cones, marked by arrestin 3 (ARR3, green), are almost absent in Cpfl1 retinae, while the overall structure of the outer nuclear layer (ONL) is preserved, compared to its massive loss in the tg(Cpfl1/Rho^−/−^) mouse model. (B) Rods (rhodopsin^+^ [RHO], green) and outer limiting membrane (membrane protein palmitoylated 5^+^ [MPP5], magenta) are intact in wild-type and Cpfl1 mice, while tg(Cpfl1/Rho^−/−^) retinae show a complete lack of rods, and the outer limiting membrane appears compromised (asterisks). (C) Müller glia are reactive in both mouse models of retinal degeneration, as observed by the upregulation of glial fibrillary acidic protein (GFAP, green), with stronger expression in a severely affected tg(Cpfl1/Rho^−/−^) mouse, contrary to wild-type retina, in which GFAP signal is restricted to astrocytes residing in the ganglion cell layer (GCL). INL, inner nuclear layer. Scale bars, 100 μm.

Moreover, Müller glia, macroglia cells that span almost the entire retina, became reactive in degenerative states identified by the upregulation of glial fibrillary acidic protein (GFAP), a phenomenon often described as gliosis. GFAP is a cytoskeleton component of the cells which, in homeostatic conditions (i.e., wild type), is expressed solely by astrocytes residing in the ganglion cell layer (GCL) (Figure 1C). In the tg(Cpfl1/Rho^−/−^) retina, GFAP was expressed throughout the Müller glia cell body, extending toward the apical processes, which was strongly pronounced when compared to the Cpfl1 retina, where GFAP expression was restricted to the level of the inner nuclear layer (INL). Despite the lack of cones in Cpfl1 mice, its OLM appeared to be continuous, while for the tg(Cpfl1/Rho^−/−^) mice the OLM appeared to be sporadically disrupted or dimmer (asterisks), as indicated by staining against the membrane protein palmitoylated 5 (MPP5/PALS1), a scaffold component of the Crumbs complex (Figure 1B).

### Survival and clustering of transplanted cells are influenced by the host’s retinal degeneration severity

A suspension of 150,000 human donor photoreceptors was administered into the subretinal space of recipient 12- to 19-week-old Cpfl1 and 15- to 18-week-old tg(Cpfl1/Rho^−/−^) mice presenting mild and severe retinal degeneration, respectively, followed by monthly intravitreal injections of the immunosuppressant triamcinolone acetonide starting on the transplantation day. Donor cells, verified by staining against mCherry expression, survived up to 26 weeks post transplantation (wpt) (latest time point analyzed) in both mildly and severely degenerated retinal microenvironments (Figure 2A). However, graft volume differed between the two degeneration states. At 26 wpt, mean graft volume corresponded to 6,349,581 μm^3^ for Cpfl1 hosts, while in tg(Cpfl1/Rho^−/−^) the mean graft volume corresponded to 2,340,832 μm^3^ (Figure 2B). Moreover, it appears that the degree of retinal degeneration severity influenced the morphology of the graft. The images selected in Figure 2A reflect retinal cross-sections with similar graft volume found for both models. In Cpfl1 hosts, donor photoreceptors tended to form multicellular clusters and were confined to a distinct retinal area, contrary to tg(Cpfl1/Rho^−/−^) hosts where they appeared mostly singularized and scattered, covering a larger part of the retina, but were rarely packed in cell clusters (Figure 2C).

**Figure 2:**
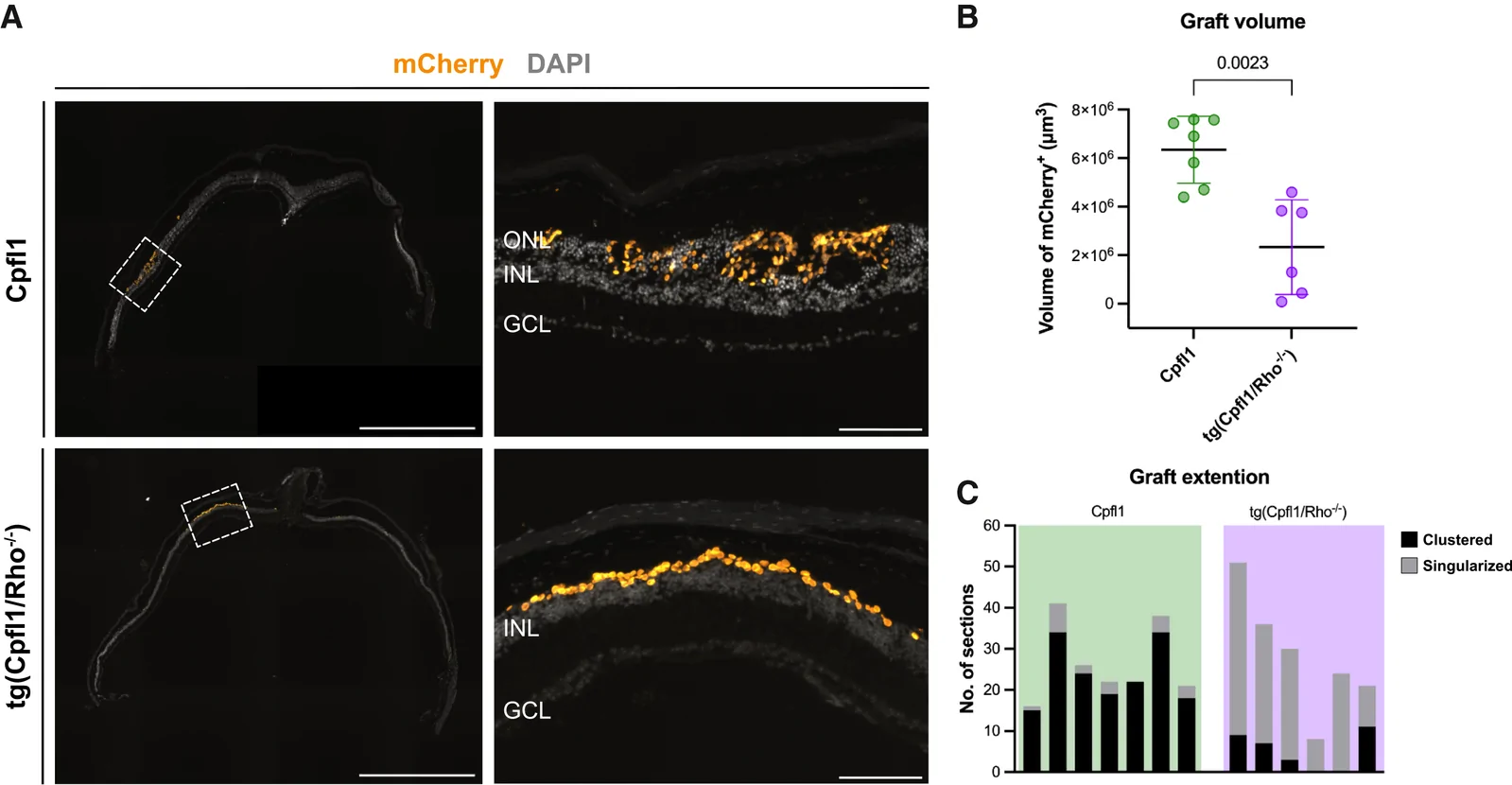
Grafts in Cpfl1 hosts show greater persistence at 26 wpt, cover less area, and appear more clustered in comparison to tg(Cpfl1/Rho^−/−^) hosts (A) Grafts (mCherry, orange) in Cpfl1 (upper row) are more clustered and spread less compared to tg(Cpfl1/Rho^−/−^) hosts (lower row). Scale bars, 1,000 μm (insets, 100 μm). (B) Graft volume is significantly larger in Cpfl1 (6,349,581 ± 1,377,204 μm^3^) compared to tg(Cpfl1/Rho^−/−^) (2,340,832 ± 1,953,060 μm^3^) hosts. Each point represents a transplanted eye. *n* = 7 (*N* = 7) for Cpfl1 and *n* = 6 (*N* = 5) for tg(Cpfl1/Rho^−/−^) hosts. Welch’s test, line at mean (SD). (C) Grafts mainly appear in multicellular clusters in Cpfl1 hosts, compared to tg(Cpfl1/Rho^−/−^) hosts, where donor photoreceptors are mostly found singularized. Each column represents a transplanted eye. ONL, outer nuclear layer; INL, inner nuclear layer; GCL, ganglion cell layer.

### Host Müller glia and transplanted photoreceptor interactions initiate at 3 wpt

Initial interactions between transplants and host cells were assessed at 3 wpt. At this time point, transplants still remained in the subretinal space in both models, with donor cells already more widely distributed in the severely degenerated tg(Cpfl1/Rho^−/−^) in comparison to mildly degenerated Cpfl1 hosts. In Cpfl1 mice, cellular “bridges” between graft and host were observed at distinct locations as the host ONL formed contact points with the graft, and Müller glia processes grew extensively into the transplant as shown by staining against glutamine synthetase (GLUL) (Figures 3A and 3A′, upper row, arrowheads) and GFAP (Figures 3B and 3B′, upper row, arrowhead). At these contact points, the OLM appeared disrupted (Figures 3A and 3A″, asterisks). In contrast, in tg(Cpfl1/Rho^−/−^) hosts, even though the graft was lying in close proximity to the remaining host INL and was apposed to GLUL^+^ (Figures 3A and 3A′, bottom row) and GFAP^+^ (Figures 3B and 3B′, bottom row) processes, these processes seemed not to grow into the graft, and the OLM appeared continuous (Figures 3A and 3A″).

**Figure 3:**
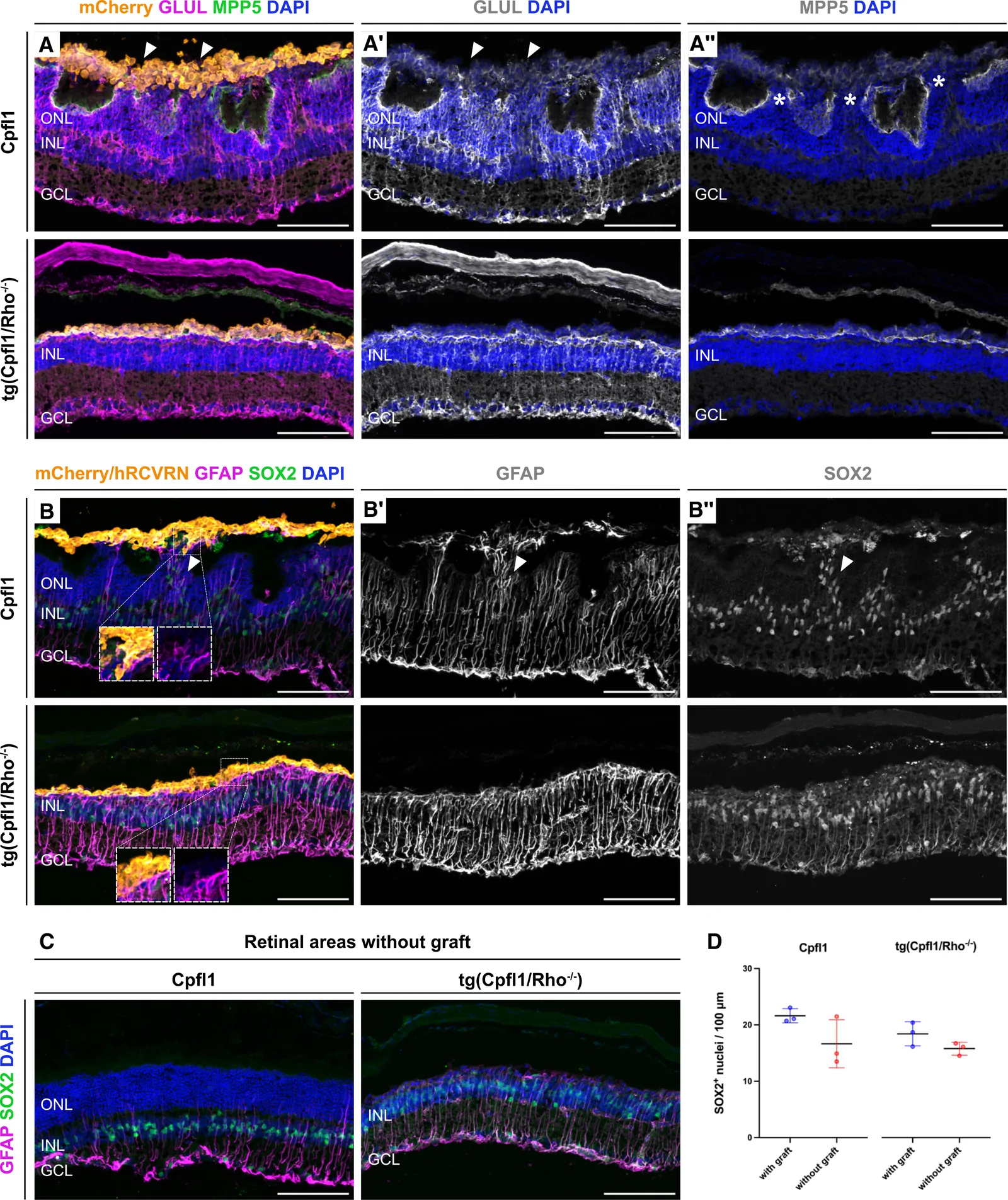
Interactions between host Müller glia and transplants at 3 wpt (A) In Cpfl1 hosts, Müller glia extend their processes (visualized by GLUL; A, magenta; A′, white) into the subretinally located graft (mCherry, orange) via distinct cellular “bridges” (arrowheads), and the outer limiting membrane (marked with MPP5; A, green; A″, white) shows disruptions at these contact points (A″, asterisks). In tg(Cpfl1/Rho^−/−^) hosts (lower row), transplanted cells also remain in the subretinal space but show no obvious interaction with host Müller glia processes, while the OLM appears undisrupted. (B) Staining for GFAP, a marker for reactive gliosis in Müller glia, confirms extensive growth of Müller glia processes into grafts in Cpfl1 but not tg(Cpfl1/Rho^−/−^) hosts (B, magenta; B′, white). Host Müller glia translocate SOX2^+^ nuclei passing the ONL toward interactive zones with transplants in Cpfl1 hosts (upper row; B, green; B″, white), which is not observed in transplanted tg(Cpfl1/Rho^−/−^) hosts. (C) The reactive profile marked by GFAP (magenta) appears increased in graft-host interactive zones (B and B′) in comparison to retinal areas without transplant (C). In retinal areas without graft, SOX2^+^ nuclei reside in the INL and do not show signs of displaced nuclei. (D) Quantification of SOX2^+^ nuclei shows an increase in areas with graft (21.63 ± 1.261 nuclei for Cpfl1, 18.40 ± 2.136 nuclei for tg(Cpfl1/Rho^-/-^)) in comparison to areas without graft (16.65 ± 4.263 nuclei for Cpfl1, 15.77 ± 1.128 nuclei for tg(Cpfl1/Rho^-/-^)). *n* = 3 (*N* = 2) per group. Wilcoxon matched-pairs signed rank, line at mean (SD). ONL, outer nuclear layer; INL, inner nuclear layer; GCL, ganglion cell layer; GLUL, glutamine synthetase; MPP5, membrane protein palmitoylated 5; RCVRN, recoverin; GFAP, glial fibrillary acidic protein; SOX2, SRY-box transcription factor 2. Scale bars, 100 μm.

Moreover, in Cpfl1 recipients, SOX2^+^ host Müller glia nuclei extended from the INL along these “bridges” toward the graft (Figures 3B and 3B″). In comparison, in contralateral areas of the same eye where there was no graft present, SOX2^+^ expression was restricted to the INL and GCL, where Müller glia and astrocytes, respectively, typically reside (Figure 3C). In addition, quantitative analysis showed a tendency for an increased number of SOX2^+^ nuclei in areas with graft compared to contralateral areas of the same retina (Figure 3D). Higher reactivity of Müller glia cells upon transplantation was also indicated by GFAP expression, as the signal appeared upregulated in the grafted areas compared to contralateral areas of the same eye (Figures 3B, 3B′, and 3C).

On the other hand, when assessing other types of host cells, for example, bipolar cells (rod bipolar cells: protein kinase C alpha [PKCa]; cone bipolar cells: secretagogin [SCGN]), amacrine cells (calretinin [CALB2]), and horizontal cells (neurofilament medium chain [NEFM]) (Figure S2), no arborizations or processes were observed to interact with donor photoreceptors at 3 wpt.

### Increased structural integration of transplants at 10 and 26 wpt

By 10 wpt, the grafts in both Cpfl1 and tg(Cpfl1/Rho^−/−^) hosts showed increased incorporation within the remaining neural retina (Figure 4A). Large parts of transplants in Cpfl1 hosts were no longer located in the subretinal space but instead were structurally integrated into the host ONL (Figure 4A, upper row, arrowheads). Host Müller glia processes—observed by staining against GLUL—enwrapped photoreceptors within the transplants (Figures 4A and 4A′, upper row, arrowheads), while the OLM—marked by MPP5—appeared disrupted in regions of graft integration (Figure 4A″, upper row, asterisks). Signs of structural integration were also observed in tg(Cpfl1/Rho^−/−^) hosts, where single donor cells showed interactions with host Müller glia processes (Figures 4A and 4A′, lower row, arrowheads), while also the OLM was no longer continuous but showed several disruptions (Figures 4A and 4A″, lower row, asterisks).

**Figure 4:**
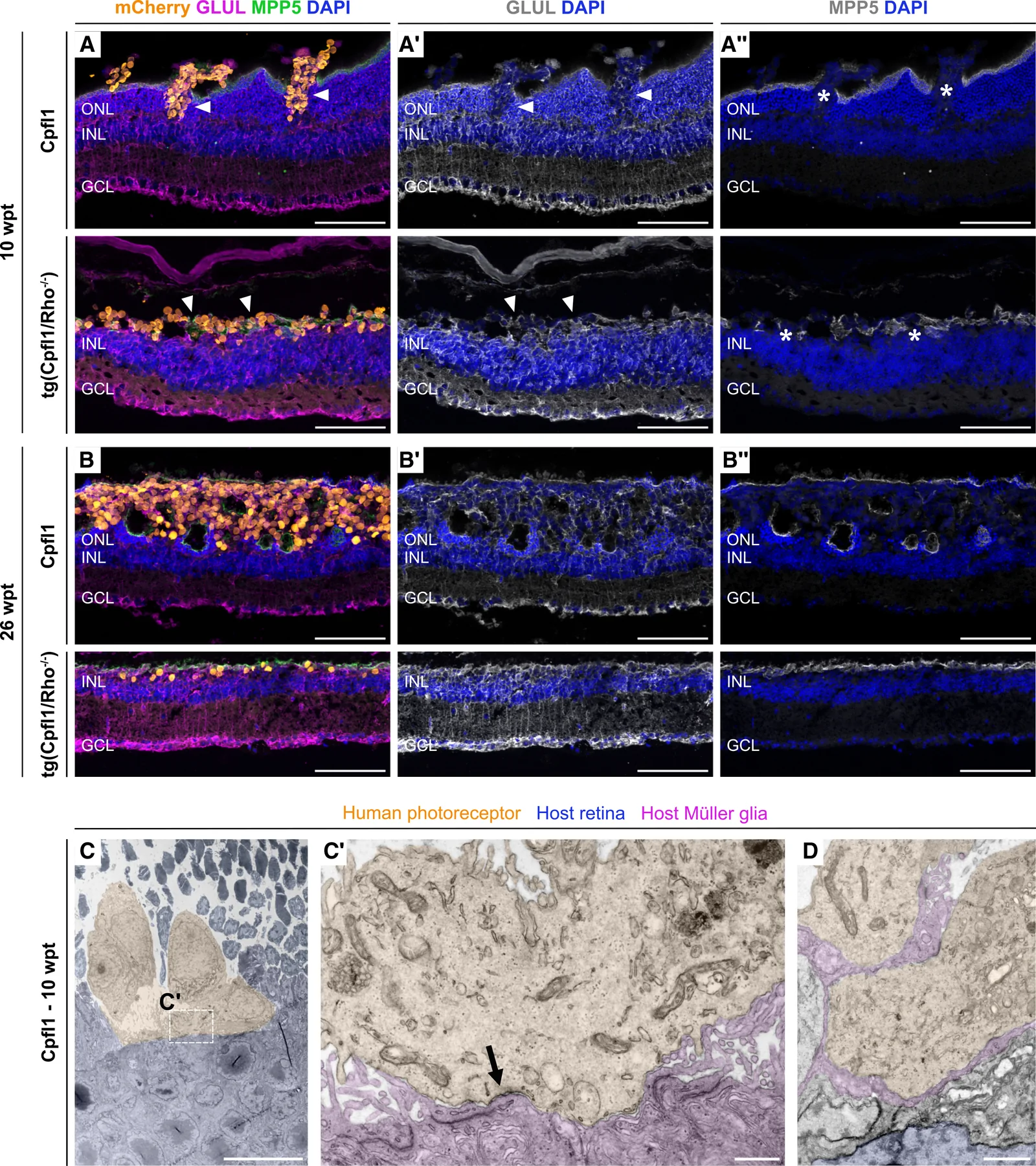
Increased integration of human photoreceptors at 10 and 26 wpt (A) At 10 wpt, human transplants (mCherry, orange) show partial incorporation into the ONL of Cpfl1 hosts (upper row), with enwrapment of donor photoreceptors by Müller glia processes marked with GLUL (A, magenta; A′, white). At the sites of partial incorporation, the OLM (MPP5; A, green, A″, white) is disrupted (marked by asterisks). First signs of enwrapment of donor photoreceptors by Müller glia processes are also observed at 10 wpt in tg(Cpfl1/Rho^−/−^) hosts (A and A′, lower row, some marked by arrowheads), while the OLM shows a disrupted appearance (A″, lower row). At 26 wpt, many donor photoreceptors appear more deeply incorporated into the retina of tg(Cpfl1/Rho^−/−^) hosts with further increased interaction with Müller glial processes (B and B′), while the OLM is mainly closed and continuous, with donor cell bodies located basally (B″, lower row). Transmission electron microscopy images show close proximity of donor photoreceptors (pseudo-colored orange) and host Müller glia (pseudo-colored magenta) in Cpfl1 hosts at 10 wpt (C, C′, and D), including formation of tight junctions (marked by an arrow). ONL, outer nuclear layer; INL, inner nuclear layer; GCL, ganglion cell layer; GLUL, glutamine synthetase; MPP5, membrane protein palmitoylated 5. Scale bars, 100 μm (A, A′, A″, B, B′, and B″), 10 μm (C), 500 nm (C′), and 1 μm (D).

Structural integration of donor photoreceptors into the host retina was confirmed for Cpfl1 hosts by ultrastructural electron microscopy analysis, where tight junctions were formed between human photoreceptors and host Müller glia (Figures 4C and 4C′, arrow), and Müller glia processes were identified in close contact to nascent inner segments of donor photoreceptors (Figure 4D).

By 26 wpt, transplants appeared to be fully incorporated within the neural retina of Cpfl1 hosts (Figure 4B, upper row), with Müller glia processes enveloping cells within the graft (Figure 4B′, upper row), while the OLM appeared resealed and continuous with cell bodies of donor photoreceptors located basally of it (Figure 4B″, upper row). Interestingly, also in tg(Cpfl1/Rho^−/−^) hosts, where there were no endogenous photoreceptors left at this time point, donor cells appeared increasingly incorporated, with several donor cells interacting with Müller glia processes (Figures 4B and 4B′, lower row). Also, the OLM appeared continuous, with donor cell bodies located basally (Figure 4B″, lower row).

### Maturation of donor cells is affected by the degenerative microenvironment

By 10 wpt, when host Müller glia largely interacted with donor photoreceptors, first signs of maturation were also distinct. Immunohistochemical staining against human-specific mitochondria (hMITO) revealed that donor photoreceptors in Cpfl1 hosts have formed single cellular substructures attached to their cell bodies, resembling inner segments typically found in maturing photoreceptors (Figure 5A, left). These bulbi co-localized with expression of recoverin (RCVRN), a pan-photoreceptor marker, and were often apically polarized into the subretinal space toward the retinal pigment epithelium (RPE). Additionally, RCVRN^+^ processes were formed by donor photoreceptors toward the host INL, reminiscent of axonal processes (Figure 5A, left, marked by arrowheads). In contrast, donor photoreceptors in tg(Cpfl1/Rho^−/−^) hosts rarely developed inner-segment- or axon-like structures at 10 wpt, with mitochondria mainly distributed throughout the cell body (Figure 5A, right). At 26 wpt, donor photoreceptors in Cpfl1 hosts showed signs of further maturation with high numbers of apical hMITO^+^ inner-segment-like protrusions and, additionally, outer-segment-like structures, as indicated by staining against opsin 1 long/medium wave sensitive (OPN1L/MW), attached to hMITO^+^ protrusions (Figure 5B, left). On the contrary, maturation of donor photoreceptors in severely degenerated hosts did not resemble the case of Cpfl1 hosts, as hMITO and OPN1L/MW^+^ extensions were rarely observed (Figure 5B, right). Quantification of the number of hMITO puncta in Cpfl1 and tg(Cpfl1/Rho^−/−^) hosts at 26 wpt reflected that a significantly larger number of transplanted photoreceptors formed inner-segment-like structures, particularly with apical polarization, within Cpfl1 retinae compared to the severely degenerated tg(Cpfl1/Rho^−/−^) retina (Figure 5C).

**Figure 5:**
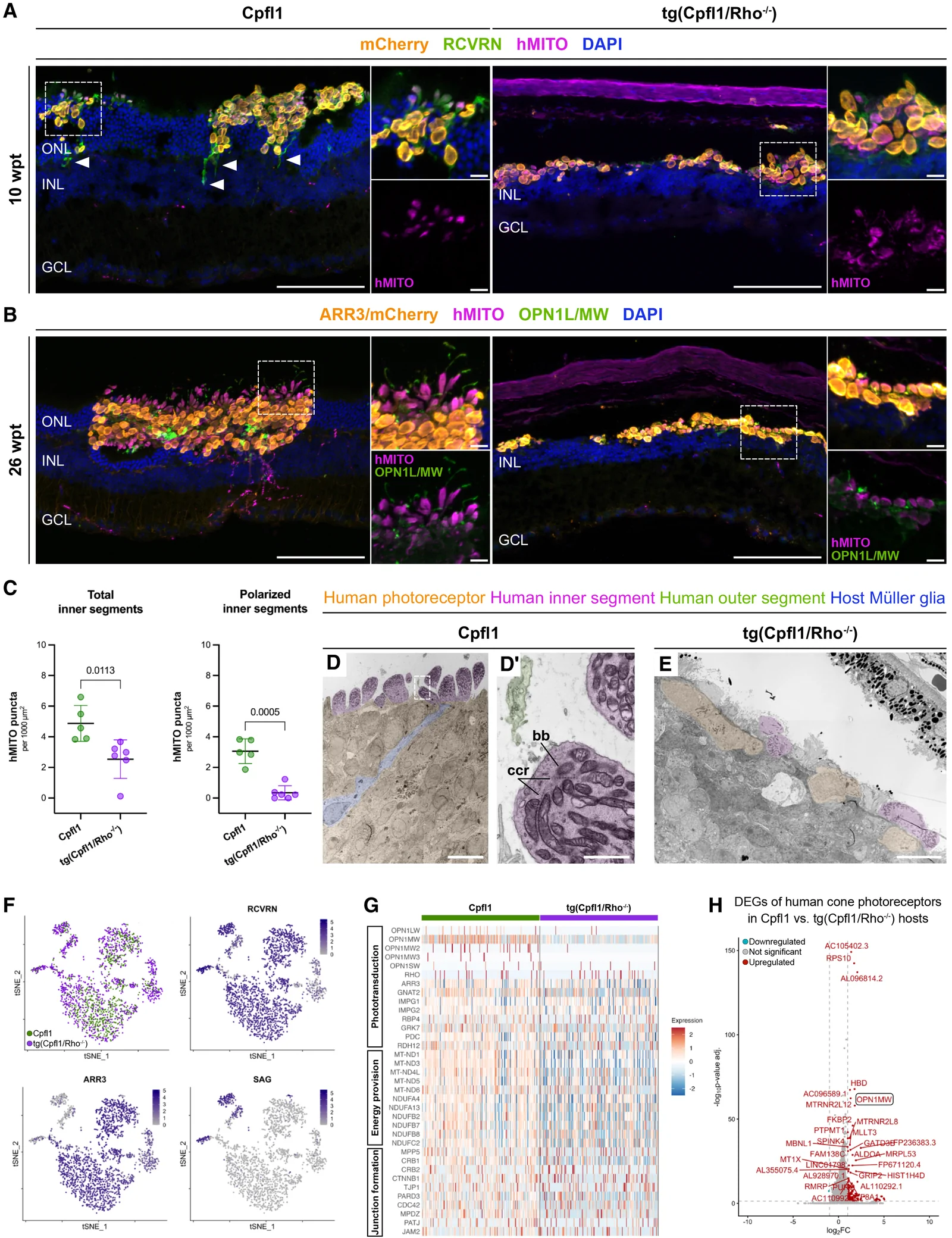
Maturation of donor photoreceptors is pronounced in Cpfl1 compared to tg(Cpfl1/Rho^−/−^) hosts (A, left) Staining against recoverin (RCVRN, green) and human-specific mitochondrial content (hMITO, magenta) reflects nascent formation and polarization of inner segments of donor photoreceptors (mCherry, orange) as well as formation of axonal processes toward the INL (marked by arrowheads) in Cpfl1 hosts at 10 wpt. (A, right) Donor photoreceptors in tg(Cpfl1/Rho^−/−^) hosts rarely show hMITO^+^ inner-segment-like protrusions or axonal processes at 10 wpt. (B, left) At 26 wpt, donor photoreceptors (ARR3/mCherry, orange) proceed to mature further in Cpfl1 hosts, generating many apical-oriented inner-segment-like protrusions (hMITO, magenta) and additionally forming outer-segment-like extensions identified by staining against cone-specific opsins (OPN1L/MW, green). (B, right) In tg(Cpfl1/Rho^−/−^), few hMITO^+^ inner-segment-like protrusions are attached to donor photoreceptors at 26 wpt, while OPN1L/MW^+^ puncta are rare and apically oriented. Scale bars, 100 μm (insets, 10 μm). (C) Quantification of hMITO^+^ puncta per 1,000 μm^2^ as a proxy for inner-segment formation. Transplanted photoreceptors in Cpfl1 hosts form significantly more total (4.878 ± 1.171 vs. 2.542 ± 1.253 puncta) as well as polarized inner segments in comparison to tg(Cpfl1/Rho^−/−^) hosts (3.062 ± 0.806 vs. 0.343 ± 0.459 puncta). Each point represents a transplanted eye. *n* = 5 (*N* = 5) for Cpfl1 and *n* = 6 (*N* = 5) for tg(Cpfl1/Rho^−/−^) hosts. Values normalized to graft area. Welch’s test, line at mean (SD). (D and D′) Transmission electron microscopy images showing a cluster of human photoreceptors (pseudo-colored orange) integrated into the retina of Cpfl1 hosts at 26 wpt with apical mitochondria-enriched inner segments (pseudo-colored magenta), nascent outer segments (pseudo-colored green), and basal body (bb) with connecting cilium rootlets (ccr). (E) Some cell bodies of donor photoreceptors (pseudo-colored orange) in tg(Cpfl1/Rho^−/−^) hosts show integration or partial integration into the retinal tissue, with nascent, apically oriented inner segments that contain few mitochondria. Scale bars, 10 μm (inset, 1 μm). (F) t-Distributed stochastic neighbor embedding (tSNE) plots from scRNA-seq analysis indicate that the donor cells are photoreceptors given the high expression of the pan-photoreceptor gene *RCVRN*, with the majority of donor cells expressing cone-specific *ARR3* and some expressing rod-specific *SAG*. (G) Heatmap reflecting expression of phototransduction-, energy-provision- and junction-formation-related genes in 200 representative donor cells isolated from Cpfl1 and tg(Cpfl1/Rho^−/−^) hosts. Overall, expression of these genes associated with maturation is higher in Cpfl1 hosts. (H) Volcano plot of differentially expressed genes (DEGs) in donor cells from Cpfl1 and tg(Cpfl1/Rho^−/−^) hosts shows upregulation of several genes associated with mature photoreceptors in Cpfl1 hosts. ONL, outer nuclear layer; INL, inner nuclear layer; GCL, ganglion cell layer; ARR3, arrestin 3; OPN1L/MW, opsin 1 long/medium wave sensitive; SAG, S-antigen visual arrestin.

Ultrastructural analysis of experimental retinae using electron microscopy at 26 wpt confirmed extensive incorporation of transplanted human photoreceptors as clusters into Cpfl1 retinae. These were accompanied by mature inner segments, which were enriched in mitochondria with a basal body (bb) neighboring to connecting cilium rootlets (ccr) as well as nascent outer segments (Figures 5D and 5D′). On the contrary, transplanted human photoreceptors in tg(Cpfl1/Rho^−/−^) retinae appeared singularized and only partially incorporated into the host tissue, albeit those were also accompanied by nascent inner segments (Figure 5E). However, inner segments appeared less structured with less mitochondria content, and no outer-segment-like structures could be detected. Generally, nuclei and inner segments of human photoreceptors were significantly larger than those of mouse photoreceptors, and human nuclei did not form the characteristic inverted architecture with centrally condensed heterochromatin seen in mouse rod photoreceptors (Figure S3A). Additionally, ultrastructural analysis of grafts at 26 wpt revealed the formation of mature ribbon synapses flanked by synaptic vesicles in Cpfl1 hosts (Figures S3A and S3A′). On the contrary, only rarely naive synaptic-like structures were detected within donor photoreceptors in tg(Cpfl1/Rho^−/−^) retinae (Figures S3B and S3B′).

### Expression of maturation markers by donor photoreceptors is increased in Cpfl1 vs. tg(Cpfl1/Rho^−/−^) hosts

To evaluate the molecular signature of human photoreceptors after transplantation into the distinct degenerative phenotypes, biopsies containing donor cells were isolated from transplanted retinae and analyzed via single-cell RNA sequencing (scRNA-seq) at 10 wpt (Figure S4A). Evaluation of key markers confirmed that donor cells represent photoreceptors (Figures 5F and S4B; exemplary photoreceptor markers: *RCVRN* and *CRX*), with the majority (ca. 80%) corresponding to cones (Figure 5F; exemplary cone marker: *ARR3*) and most remaining cells expressing rod genes (Figure 5F; exemplary rod marker: *SAG*). Extremely rarely, Müller glia cells or bipolar cells showed up in the donor cell population, as examined by the expression of the markers vimentin (*VIM*) and carbonic anhydrase 10 (*CA10*), respectively (Figures S4C and S4D). This confirms that Müller glia or bipolar cell signals observed in close contact or within the transplants derived from the host, and were not introduced by the transplanted cell suspension. Moreover, expression of genes involved in the processes of phototransduction, energy provision, and junction formation—all characteristics of a mature photoreceptor—was higher in transplants within Cpfl1 mice compared to tg(Cpfl1/Rho^−/−^) hosts (Figure 5G; heatmap represents gene expression of 200 donor photoreceptors downsampled per condition), underlining an overall improved maturation status of donor photoreceptors in the milder degenerative microenvironment. Indeed, an exploratory comparison of differentially expressed genes (DEGs) of the cone fraction from the two hosts revealed that *OPN1MW* was one of the highest upregulated genes of donor photoreceptors within Cpfl1 recipients (Figure 5H).

### Clustered donor cells do not show improved incorporation and maturation in tg(Cpfl1/Rho^−/−^) hosts

Donor photoreceptors showed a wider distribution and singularization after transplantation into tg(Cpfl1/Rho^−/−^) mice, while in corresponding Cpfl1 hosts donor cells formed larger cell clusters with signs of improved integration and maturation. To evaluate whether the number of clustered grafts can be increased and thus interactions with host Müller glia as well as integration and photoreceptor maturation can be improved, a suspension of 500,000 donor cells (instead of 150,000 cells) was administered in both models and assessed at 10 wpt. Even though donor cells had an increased propensity of forming clusters in the tg(Cpfl1/Rho^−/−^) recipients, they appeared mostly isolated from the rest of the host retina (Figures 6A and 6B, right). Compared to the well-structured and polarized inner segments in Cpfl1 hosts, indicated by hMITO and hRCVRN expression, such bulbi were still rarely observed in the severe model of retinal degeneration (Figure 6).

**Figure 6:**
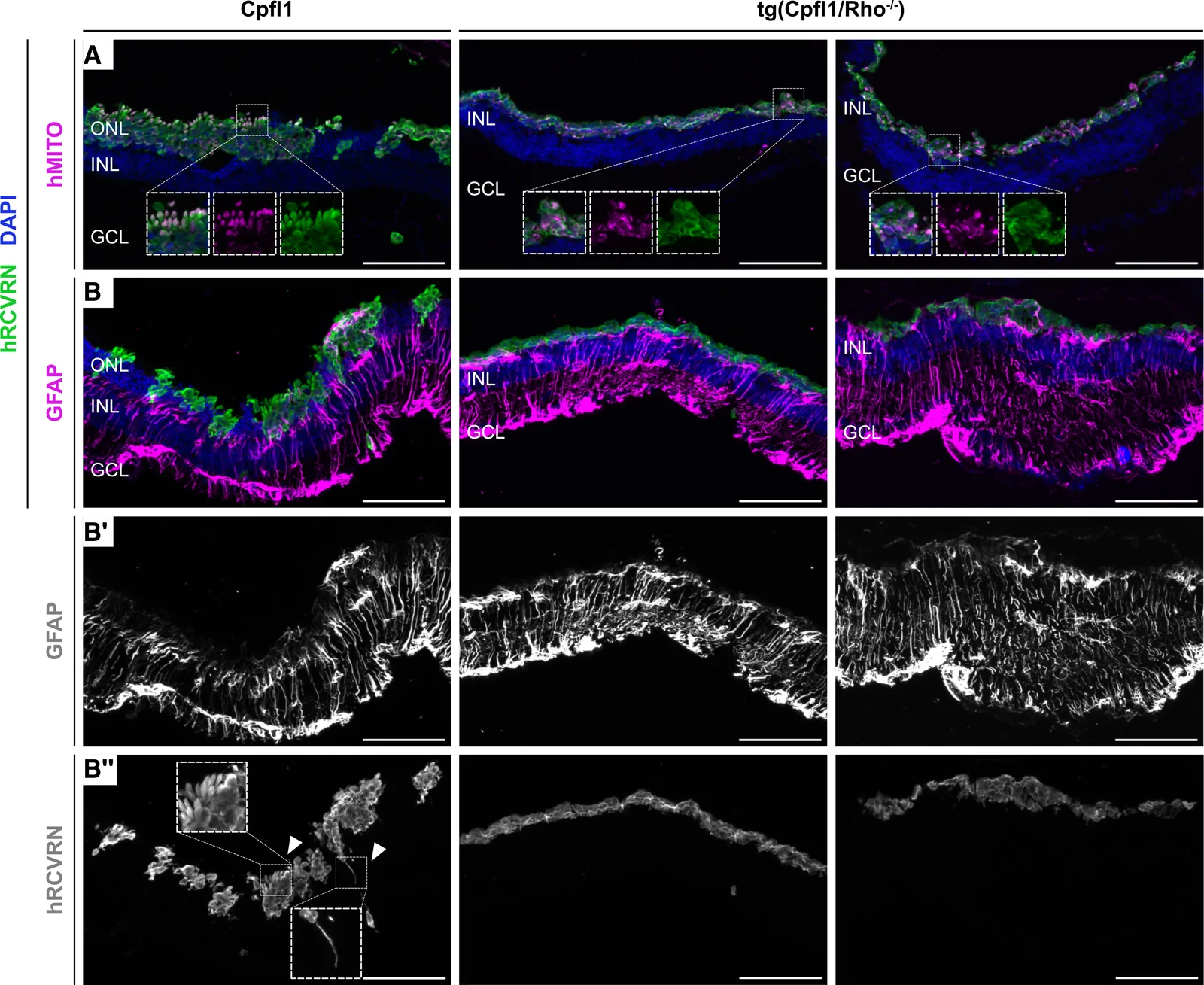
Clustering of transplanted photoreceptors does not suffice for their maturation (A) Following transplantation of 500,000 cells, donor photoreceptors (hRCVRN, green) are clustered in both Cpfl1 and tg(Cpfl1/Rho^−/−^) recipients at 10 wpt, but hMITO^+^ (magenta) inner segments are formed and apically polarized much more in the Cpfl1 retina. (B) Host Müller glia (marked with GFAP; B, magenta; B′, white) interact vastly with the graft in Cpfl1 contrary to tg(Cpfl1/Rho^−/−^) hosts. (B″) Donor photoreceptors expressing hRCVRN (white) generate apical inner segments and basal axonal processes in Cpfl1 but not tg(Cpfl1/Rho^−/−^) hosts. ONL, outer nuclear layer; INL, inner nuclear layer; GCL, ganglion cell layer; hRCVRN, human-specific recoverin; hMITO, human-specific mitochondrial content; GFAP, glial fibrillary acidic protein. Scale bars, 100 μm.

Moreover, interactions between host Müller glia and donor photoreceptors still remained sparse in the tg(Cpfl1/Rho^−/−^) mouse (Figures 6B and 6B′, right). On the contrary, following transplantation of 500,000 cells, donor photoreceptors had partially incorporated into the host ONL of Cpfl1 hosts and were widely enwrapped by host Müller glia processes (Figures 6B and 6B′, left). Staining against hRCVRN highlighted the mature structure of donor photoreceptors in Cpfl1 hosts, as both segments and neurites could be found, whereas such structures were mainly absent from tg(Cpfl1/Rho^−/−^) hosts (Figures 6B and 6B″). These findings further support the correlation of donor-host interactions with their subsequent incorporation and maturation and that the morphology of the graft—whether donor photoreceptors were in clusters or singularized—did not seem to impact these processes in tg(Cpfl1/Rho^−/−^) hosts.

### Transplantation at an earlier stage of progressive degeneration results in improved incorporation and maturation of donor photoreceptors as well as remodeling of host bipolar cells

Hypothesizing that the retina of Cpfl1 hosts might provide a better scaffold due to the remaining ONL, 150,000 donor cells were transplanted into 4-week-old tg(Cpfl1/Rho^−/−^) mice—henceforth referred to as tg(Cpfl1/Rho^−/−^)^early^—a time point when approximately 90% of endogenous photoreceptors, and thus an ONL, are still present (Figure S5). Qualitative immunohistological assessment of grafts at 26 wpt in tg(Cpfl1/Rho^−/−^)^early^ hosts revealed a clustering phenotype consisting of mature photoreceptors similar to those observed in Cpfl1 hosts (Figure 7A). Inner segments, defined by staining against hMITO and hRCVRN, appeared analogous in shape to those formed in Cpfl1 hosts and were largely polarized toward the subretinal space/RPE (Figure 7B). Host Müller glia GLUL^+^ processes enveloped donor photoreceptors, and a nascent OLM was formed (MPP5) (Figure 7C). Interestingly, endogenous photoreceptors, of which 90% were still present at the time of transplantation, were completely lost in tg(Cpfl1/Rho^−/−^)^early^ hosts at 26 wpt despite integration and maturation of donor photoreceptor clusters.

**Figure 7:**
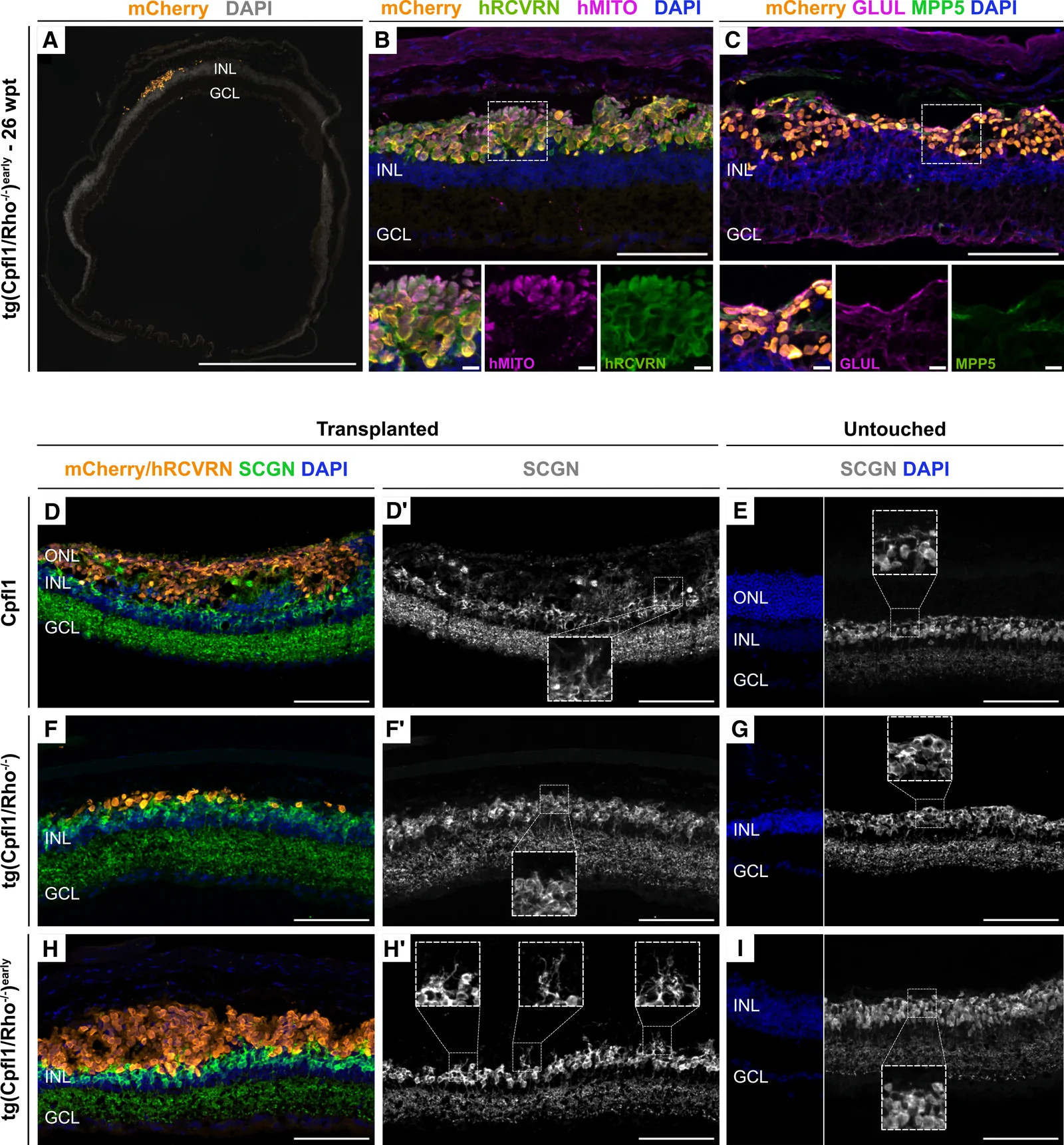
Photoreceptor transplants in tg(Cpfl1/Rho^−/−^)^early^ hosts show improved clustering and maturation and also incite dendrite regrowth of host bipolar cells (A) Donor photoreceptors (mCherry, orange; hRCVRN, green) appear clustered in tg(Cpfl1/Rho^−/−^)^early^ hosts and (B) generate polarized inner segments (hMITO, magenta). (C) Host Müller glia (GLUL, magenta) envelop the donor cells in tg(Cpfl1/Rho^−/−^)^early^ hosts, and a nascent outer limiting membrane (MPP5, green) is formed. (D) In Cpfl1 hosts, cone bipolar cells grow dendrite processes (SCGN, green; in D′, white) toward/into the transplant (mCherry/hRCVRN, orange). (E) Apical dendrite processes, though shorter, are also present in the untouched Cpfl1 retina. (F, F′, and G) tg(Cpfl1/Rho^−/−^) retinae do not form dendrite outgrowths, neither with nor without photoreceptors transplanted at a late degeneration stage. (H, H′, and I) While host cone bipolar cells in untouched retinae retract their dendrites in tg(Cpfl1/Rho^−/−^)^early^ at 26 wpt, SCGN^+^ dendrite outgrowths are visible in areas containing transplants. ONL, outer nuclear layer; INL, inner nuclear layer; GCL, ganglion cell layer; hRCVRN, human-specific recoverin; hMITO, human-specific mitochondrial content; GLUL, glutamine synthetase; MPP5, membrane protein palmitoylated 5; SCGN, secretagogin. Scale bars, 100 μm (insets, 10 μm).

In Cpfl1 hosts, cone bipolar cells (marked by SCGN) grew dendritic processes deeply into structurally integrated transplants (Figures 7D and 7D′), with extended length in comparison to cone bipolar cells in non-transplanted retinae (Figure 7E). In age-matched untouched tg(Cpfl1/Rho^−/−^) and tg(Cpfl1/Rho^−/−^)^early^ retinae (i.e., 42 and 30 weeks old, respectively), host bipolar cells had fully retracted their dendrites, most likely due to complete degeneration of the host ONL (Figures 7G and 7I). In tg(Cpfl1/Rho^−/−^) retinae transplanted at late stage, growth of cone bipolar dendritic processes remained low in regions with or without donor cells (Figures 7F and 7F′). In contrast, in tg(Cpfl1/Rho^−/−^)^early^ hosts, bipolar cells underwent remodeling, and their dendrites regrew toward the graft (Figures 7H and 7H′), similarly to Cpfl1 hosts.

## Discussion

Preclinical studies from several laboratories have provided the first evidence for successful transplantation of human photoreceptors isolated from PSC-derived retinal organoids, including some functional improvements in mouse models of photoreceptor loss.^13^ Retinal degenerative diseases are characterized by extremely high genetic (hundreds of genes with disease-causing mutations identified)^14^^,^^15^ and phenotypic heterogeneity, with diverse complex pathologies described, ranging from early to late onset and slow to fast photoreceptor death. While photoreceptor replacement represents a potential generalized disease-agnostic treatment approach, insights into whether and to what extent such highly diverse disease environments affect photoreceptor transplants have not been systematically assessed. In this study, we utilized two mouse models showing distinct levels of photoreceptor degeneration to investigate in which ways the underlying retinal degenerative status influences transplanted human photoreceptors in regard to survival, structural integration, and maturation by employing histological, ultrastructural, and transcriptome analysis. We observed that photoreceptor transplants in the severely degenerated retina survive, cluster, and mature to a much lesser extent than in the milder model of retinal degeneration. Interestingly, transplantation into the severe mouse model at an early disease stage appears to allow structural integration and maturation of transplants despite continuous loss of endogenous photoreceptors.

Human donor photoreceptors, enriched from iPSC-derived retinal organoids at D200, were subretinally transplanted into mild (Cpfl1) or severe (tg(Cpfl1/Rho^−/−^)) degenerative hosts. While in both disease environments donor cells showed long-term survival for up to 26 wpt, the latest time point examined, grafts showed significant differences in their distribution within the subretinal space: in Cpfl1 recipients donor photoreceptors mainly form multicellular clusters, whereas in tg(Cpfl1/Rho^−/−^) mice they cover a larger part of the retina but appear singularized. Quantification of combined nuclei volume of donor cells within the two mouse models revealed significantly larger volumes in Cpfl1 hosts arguing for improved long-term graft persistence, thus implying better survival. Singularized distribution was also observed by Collin and colleagues when transplanting human ESC-derived CRX^+^ cells into the severely degenerated rd1 mouse line.^1^ On the contrary, in a study by Ribeiro and colleagues,^4^ in which 500,000 human cone photoreceptors were transplanted into rd1 mice, donor cells appear clustered. Similar findings were observed in research by Procyk and colleagues,^7^ in which the Aipl1^−/−^ mouse model—presenting complete ONL degeneration—was used. Indeed, larger grafts were also observed in our study after transplantation of 500,000 cells into the late-stage mouse model; however, this seemingly does not improve the incorporation and maturation of donor photoreceptors.

When comparing graft morphologies between models at the three different time points examined—3, 10, and 26 wpt—it is apparent that donor cells, over time, cluster more in Cpfl1 hosts. Rarely, within the grafted area of the retina, rosette-like structures were observed to be formed by the underlying host ONL at 26 wpt. This feature of photoreceptor reorganization resembles tubulations observed in retinal degeneration speculated to form upon sustained photoreceptor or RPE damage.^16^ In the context of photoreceptor replacement, the cystic spaces or tubulations adjacent to the transplant could be attributed to the local death of endogenous photoreceptors due to the absence of direct contact with the RPE. However, the exact reason behind their formation is currently unknown. Donor cells in tg(Cpfl1/Rho^−/−^) hosts remain rather dispersed and stretched throughout the length of the retina. We hypothesize that the interphotoreceptor matrix, the extracellular matrix coating the subretinal space, might also undergo remodeling proportional to the extent of photoreceptor degeneration. This could potentially influence the clustering effect from the moment of the surgical intervention, as the weakened interface between the host neural retina and RPE could result in the broader dispersion of the injected donor cells. This is further supported by our results regarding transplantations in the early degeneration stage of tg(Cpfl1/Rho^−/−^) hosts, where only 10% of endogenous photoreceptors are lost at the time of transplantation and where the donor photoreceptors remain clustered at 26 wpt. Host cells might also participate in these morphological differences, as at 3 wpt they contact the graft in Cpfl1 hosts, and the graft from that point onward becomes more compacted and spatially restricted. Notably, the delivery of more donor cells in transplantations, 500,000 vs. 150,000, seems to potentiate more interactions between them, a parameter that is tunable. Nevertheless, the absolute number of contact points between host neural retina and graft needs to be assessed in future studies.

Interestingly, scRNA-seq analysis revealed that more cones than rods are present in the graft population at 10 wpt, a selectivity which remains to be investigated, given that a similar proportion of cones and rods are observed in retinal organoids produced by our standardized protocol.^11^ Additionally, donor cones predominantly differentiated toward the medium-wavelength cone subtype. Adjusting the number and proportion of cell types of administered cells are elements for consideration in tailoring potential cell therapies to the needs of patients (e.g., patients with achromatopsia would require a cone-rich transplantation, while retinitis pigmentosa patients would require rod-rich transplants, and those at later stages with complete blindness would require both photoreceptor types).

At 3 wpt, host Müller glia expand their processes toward the graft, co-aligning with our previous observations when transplanting human iPSC-derived cones into Cpfl1 hosts,^2^ and translocate some nuclei from the INL to the ONL. An increase of SOX2^+^ nuclei is also found in transplanted areas compared to non-transplanted areas of the same retina, an event that is not model-specific, occurring in Cpfl1 and tg(Cpfl1/Rho^−/−^) hosts. Such phenomena could indicate glial proliferation, a process that has been associated with interkinetic nuclear migration observed during retinal regeneration of zebrafish.^17^ Interestingly, transcriptomic data by Sudharsan and colleagues reveal an upregulation of pathways related to proliferation and DNA synthesis in host glial cells after transplantation of photoreceptor precursors into canine retinae.^18^ Nevertheless, it remains to be clarified whether the observed increased number and translocation of SOX2^+^ nuclei is related to proliferation and whether it represents interkinetic nuclear migration or a whole cell body migration event. Notably, even though Müller glia pretransplantation appear to have a reactive state in both Cpfl1 and tg(Cpfl1/Rho^−/−^) retinae, indicated by the observed GFAP upregulation, this does not appear to be inhibitory toward host-graft interactions, as often suggested due to the formation of a gliotic seal seen in retinal degeneration.^19^^,^^20^^,^^21^ Furthermore, although Cpfl1 retinae are characterized by a continuous OLM, this structure does not hinder graft incorporation as it is transiently interrupted and then resealed, reflecting an extent of plasticity as previously reported.^2^^,^^18^ Overall, these results suggest that host Müller glia act in a dynamic manner upon transplantation, being the first responders in mediating interactions with the graft and initiating graft incorporation. The grafts appear fully integrated by 26 wpt in Cpfl1 hosts and partially incorporated into tg(Cpfl1/Rho^−/−^) hosts. Importantly, the features discriminating human donor photoreceptors from mouse host photoreceptors, such as their significant larger nucleus and inner-segment size and a non-inverted nuclear structure,^2^^,^^7^ support the paradigm of a true incorporation event rather than a potential cytoplasmic material transfer phenomenon, observed so far only in intraspecies transplantations.^22^^,^^23^^,^^24^^,^^25^ Furthermore, examination of species-specific transcript expression by us (K.T., unpublished data) and others^18^ revealed that the vast majority of transcripts (approximately 70%–80%) within reporter-labeled cells isolated from experimental retinae were of human origin, while the remaining transcripts could not unambiguously be assigned to a specific species, strongly arguing against material transfer events. Taking into consideration that grafts at 3 wpt in the severely degenerated hosts remained isolated from the rest of the neural retina, these observations suggest delayed incorporation and, as such, differential incorporation kinetics into the host. Understanding how early host-graft interactions potentiate the long-term graft survival and functional integration efficiency might be key for the treatment of patients with late-stage pathology.

Differences were also observed regarding the maturation of transplanted photoreceptors within the two degeneration models at the phenotypic as well as molecular level. We observed formation of polarized inner- and outer-segment-like structures in the transplanted photoreceptors in Cpfl1 hosts, a morphology closely reminiscent of a mature photoreceptor. On the contrary, less and disorganized segments are found in tg(Cpfl1/Rho^−/−^) hosts where both hMITO^+^ and OPN1L/MW^+^ signals were diffusely distributed within the transplants, underlining an influence of retinal degeneration severity on *in vivo* maturation of donor photoreceptors. The morphological differences were recapitulated by scRNA-seq analysis showing increased expression of genes related to maturation of donor photoreceptors within Cpfl1 hosts including phototransduction, energy metabolism, and junction formation. Exploratory differential expression analysis between the two disease models further punctuated the higher expression of inner- and outer-segment-specific genes in donor photoreceptors of Cpfl1 hosts. As samples for this ranking analysis were pooled *a priori*, discrimination of individual biological replicates is not possible, and therefore caution is required in interpreting the corresponding *p* values. Others have reported partial functionality rescue in severely degenerated retinae,^3^^,^^4^^,^^7^^,^^26^^,^^27^ despite donor photoreceptors providing also fewer signs of full maturation, such as the ones observed here within the mild model. It is therefore of interest to assess to what extent graft maturation influences functionality post transplantation, a correlation analysis that was beyond the scope of this study.

Advanced maturation of donor cells within Cpfl1 hosts might result from the closer/stronger interaction with host Müller glia cells (see above). Indeed, in retinal development and physiology, Müller glia partake in assembly as well as proper function and integrity of photoreceptors.^28^^,^^29^ Thus, deciphering the cellular and molecular factors facilitating interactions between host Müller glia and donor photoreceptors might identify targets for improving graft integration in future studies. Interestingly, increasing the number of donor photoreceptors to 500,000 cells, despite seemingly potentiating more contacts between the graft and host tissue in the tg(Cpfl1/Rho^−/−^) hosts, appeared to be not sufficient for further photoreceptor maturation. However, while interactions with Müller glia cells emerge to be a main factor for donor photoreceptor polarization and maturation, other physiological components might also play important roles in this process. This might entail the surrounding subretinal matrix, as transplanted photoreceptors at the tg(Cpfl1/Rho^−/−^)^early^ stage showed better morphology including inner-segment formation. Other factors might include synapse formation between donor photoreceptors with second-order neurons or interactions with RPE cells. The latter is of importance, as certain pathologies are also characterized by RPE degeneration, which could in turn damage further transplanted photoreceptors, as maintenance and health of light-sensitive cells highly depends on the integrity of this epithelium. By investigating, therefore, different parameters of the diseased retinal microenvironment, we hope to improve the degree of incorporation and maturation of donor photoreceptors following transplantation into different states of retinal degeneration.

Thus, these findings provide evidence that the development and physiological status of photoreceptor transplants is significantly influenced by the recipient environment and should be taken into account in the development of photoreceptor replacement therapies. Nevertheless, the exact degeneration extent of the diseased retina, and therefore the therapeutic window that would be amenable for successful cell replacement therapy, needs to be further defined. While early-stage interventions might be unlikely to be adopted in the clinic, as surgical intervention often triggers inflammation and might exaggerate the death of the remaining host photoreceptors,^18^ this approach could be perhaps applicable for conditions characterized by early significant function loss despite the morphological integrity of residual photoreceptors. Additionally, a genetic correctional approach of the disease-causing mutations in patients would be required in order to achieve autologous transplantations and progress toward the clinic.^30^

## Materials and methods

### Maintenance of human iPSCs and differentiation in retinal organoids

The iPSC AAVS1:CrxP H2BmCherry line^2^^,^^10^ used for the generation of HROs was kindly provided by Olivier Goureau (Institute de la Vision, Paris, France) and maintained at the Stem Cell Facility of the Center for Molecular and Cellular Bioengineering (CMCB, TU Dresden). iPSCs were cultured in mTeSR 1 medium (STEMCELL Technologies, Cologne, Germany, #85850) on vitronectin-coated plates (Gibco, Dreieich, Germany, #A400458) and passaged using ReLeSR (STEMCELL Technologies, #100-0484) at 37°C. Differentiation of iPSCs into HROs was performed using a previously optimized protocol.^31^ In brief, on D0, undifferentiated iPSCs at 60%–80% confluency were washed twice with phosphate-buffered saline (PBS) and incubated with ReLeSR for 2 min at 37°C. Cells were resuspended in DMEM/F-12 + GlutaMAX (Gibco, #31331028), collected using wide-bore pipette tips, and centrifuged for 3 min at 700 rpm at room temperature (RT). The cell pellet was placed in ice and resuspended in N2B27 medium (1:1 DMEM/F-12 + GlutaMAX: Neurobasal medium [Gibco, #21103-049], 1% B27 with vitamin A [Gibco, #17504044], 0.5% N2 [Gibco, #17502048], 1% penicillin-streptomycin [Sigma-Aldrich, Taufkirchen, Germany, #P0781], and 50 μM β-mercaptoethanol [Gibco, #31350010]). Growth-factor-reduced Matrigel (Corning, Berlin, Germany, #354230) was added to the cell solution and allowed to polymerize for ca. 7 min. The Matrigel was dispersed in N2B27 medium, and aggregates were seeded in low-attachment 6-well plates (Thermo Scientific, Dreieich, Germany, #174945). Fresh medium was added on D3, and on D5 neuroepithelial cysts were transferred to Matrigel-coated 6-well plates at a 1:2 ratio. Fresh medium was added on D7 and D9, followed by a full medium change on D11. On D13, adherent cysts were washed twice with DMEM/F-12 and detached using Dispase (STEMCELL Technologies, #07923). After a 15-min incubation at 37°C, the cysts were mechanically detached and collected into 15-mL tubes. After two washing steps with DMEM/F12, the cysts were transferred to ultra-low attachment Petri dishes (Thermo Scientific, #174945) containing B27 medium (DMEM/F12 GlutaMAX, 2% B27 without vitamin A [Gibco, #12-587-010], 1% penicillin-streptomycin, 1% non-essential amino acids [Gibco #11140035], and 0.1% amphotericin B [Gibco, #15290026]). A half medium change was done the day after and then every 2–3 days. On D25, the medium was replaced with B27/FBS medium (B27 media with 10% fetal bovine serum [Gibco, #A5256801]) plus 0.3 μM EC23 (Tocris, Wiesbaden-Nordenstadt, Germany, #4011). Between D30 and D40, retinal epithelial evaginations were manually isolated using surgical forceps. On D100, the HROs were cultured in RM2 medium (DMEM/F12 GlutaMAX, 1% N2, 10% FBS, 1% penicillin-streptomycin, and 0.1% amphotericin B) plus 0.3 μM EC23. After 20 days (D120), EC23 was completely removed and medium was changed every 2–3 days until D200.

### FACS of mCherry^+^ cells

D200 HROs were dissociated into a single-cell suspension following our established protocol.^12^ In brief, the HROs were incubated with 20 U/mL papain for 1.5 h, then mechanically triturated using a narrow fire-polished glass pipette and processed according to the manufacturer’s instructions (Papain Dissociation System, Worthington Biochemical, Lakewood, NJ, USA). The cell pellet was then resuspended in magnetic-activated cell sorting (MACS) buffer (0.5% BSA and 2 mM EDTA in PBS), which was filtered through a 35-μm mesh, reaching a final concentration of ca. 7 million cells/mL. Samples were stored on ice and then sorted with either AriaFusion, AriaII, or AriaIII, using a 100-μm nozzle at 4°C. Before isolation of mCherry^+^ cells based on the fluorescent intensity, debris and doublets were removed by adjusting the forward-scatter and side-scatter areas. 4′,6-Diamidino-2-phenylindole (DAPI) (AppliChem, Darmstadt, Germany, #A1001.0010) staining was applied to remove dead cells.

### Animals

Adult Cpfl1-mutant (12–19 weeks of age), tg(Cpfl1/Rho^−/−^)^early^-mutant (4 weeks of age), and tg(Cpfl1/Rho^−/−^)-mutant (15–18 weeks of age) mice were used as recipients for cell-suspension transplantation. Only female mice were used for transplantations to reduce potential sex-specific variability. Depending on the experimental setup, one or both eyes were transplanted in each mouse. In the 150,000-cells/μL transplantations, the sample size (*n* = number of eyes; *N* = number of animals) for the immunohistochemical analysis at 3 wpt was *n* = 3 (*N* = 2) for Cpfl1 and *n* = 3 (*N* = 2) for tg(Cpfl1/Rho^−/−^); for the 10-wpt analysis, this was *n* = 6 (*N* = 6) for Cpfl1 and *n* = 8 (*N* = 8) for tg(Cpfl1/Rho^−/−^); and for the 26-wpt analysis, *n* = 7 (*N* = 7) for Cpfl1, *n* = 6 (*N* = 4) for tg(Cpfl1/Rho^−/−^)^early^, and *n* = 6 (*N* = 5) for tg(Cpfl1/Rho^−/−^). For the electron microscopy analysis at 26 wpt, the sample size was *n* = 2 (*N* = 2) for Cpfl1 and *n* = 1 for tg(Cpfl1/Rho^−/−^). For the sequencing analysis at 10 wpt, the sample size was *n* = 3 (*N* = 3) for Cpfl1 and *n* = 3 (*N* = 3) for tg(Cpfl1/Rho^−/−^). For the immunohistological analysis at 10 wpt of 500,000-cells/μL transplantations, the sample size was *n* = 6 (*N* = 3) for Cpfl1 and *n* = 6 (*N* = 3) for tg(Cpfl1/Rho^−/−^). Additionally, untouched wild-type C57BL/6JRj mice (17 weeks of age) were used for histological analysis (*n* = 3, *N* = 2). The colonies were maintained in the CRTD animal facility on a 12-h light/12-h dark cycle with *ad libitum* access to food and water. After intervention, gel food was provided to the animals, and regular health checkups were performed.

### Optical coherence tomography

The setup for optical coherence tomography (OCT) was used as described previously.^32^ In brief, mice were anesthetized with an intraperitoneal injection of a mixture of 100 mg/mL ketamine (Serumwerk Bernburg, Bernburg, Germany, #13690.00.00) and 1 mg/mL medetomidine (Orion Pharma, Hamburg, Germany, #32457.00.00), and a drop of 2.5% phenylephrine/0.5% tropicamide solution (University Clinic Pharmacy, Dresden, Germany) was added to the eye for 2–3 min to dilate the pupil. The excess was removed, and hydrating gel (Bausch & Lomb, Berlin, Germany, #40612.00.00) was added before placing a glass coverslip on the cornea. The OCT scanner was placed in parallel to the eye, and cross-sections of the central retina were acquired every 4 μm. After the final scan, anesthesia was immediately reversed by intraperitoneal injection of 5 mg/mL atipemazole hydrochloride (Antisedan; Orion Pharma, Hamburg, Germany, #23554.00.00). Recordings were processed in ImageJ (NIH).

### Transplantations

Post FACS, mCherry^+^ cells were resuspended in MACS buffer (150,000 cells/μL or 500,000 cells/μL), and 1 μL was delivered into the subretinal space of anesthetized recipient mice with a Hamilton syringe mounted to a micromanipulator as described in Tessmer et al.^12^ Following transplantation and on a monthly basis, 1 μL of triamcinolone acetonide suspension (80 μg/μL in NaCl; University Clinic Pharmacy, Dresden, Germany) was delivered intravitreally. Anesthesia was immediately reversed.

### Immunohistochemistry and immunocytochemistry

For immunohistochemistry, transplanted mice were euthanized, and eyes were enucleated and fixed in 4% paraformaldehyde in PBS for 1 h at 4°C. The muscle, cornea, lens, and optic nerve were dissected away, and the eyes were cryopreserved in a 30% (w/v) sucrose solution overnight at 4°C. The tissue was then embedded in optimal cutting medium NEG50 (Epredia, Dreieich, Germany, #6502), and 12-μm cross-sections were collected on SuperfrostPlus Adhesion Microscope Slides (Epredia, #J1800AMNZ) and dried in a heating platform. Slides were first hydrated with PBS for 30 min and then incubated with blocking solution (0.3% Triton X-100, 1% BSA, and 5% donkey solution in PBS) for 1 h at RT before incubation with primary antibodies overnight at 4°C. The next day, 3 × 10-min washes with PBS were performed, followed by a 90-min incubation with respective secondary antibodies and another 3 × 10-min washes with PBS. Lastly, the slides were dried and mounted in Aqua-Poly/Mount (Polysciences, Hirschberg an der Bergstrasse, Germany, #18606-20) and allowed to fully dry. Primary and secondary antibodies used are listed in Table S1 and Table S2, respectively. All secondary antibodies were used in a 1:1,000 dilution.

### Image acquisition and image analysis

Samples were imaged using a Zeiss (Germany) Apotome Imager Z2. For evaluation of graft size, full AxioScan images were acquired with a Zeiss AxioScan.Z1. Images were processed using ImageJ2 (v.2.3.0/1.53s) and Affinity Designer (v.1.10.4). Images shown were generated using the Max Intensity Projection algorithm on *z* stacks of 1-μm intervals in ImageJ2. The graft volume of each transplanted eye was quantified based on the fluorescent CRX-mCherry signal post immunohistochemistry using the formula sum of graft area (μm) × 12 μm × 4. In brief, the summed graft area of every fourth serial retinal cross-section throughout each transplanted eye was measured, multiplied by 12 μm to correct for the thickness of the tissue (as the acquired retinal sections were 12 μm thick), and lastly multiplied by 4 to correct for the unmeasured serial sections. Maturation capacity of donor photoreceptors was assessed in grafts that were in their entirety incorporated within the host ONL (in the cases of Cpfl1 hosts) or in contact with the remaining neural retina (in the cases of tg(Cpfl1/Rho^−/−^) hosts). To measure the number of inner segments formed in grafts, hMITO signal—localized in human mitochondria—was used as a proxy. The samples from the graft volume analysis in Figure 2B were used also for the hMITO quantifications in Figure 5C. Because in two transplanted Cpfl1 eyes small parts of the graft were found as floating clusters in the subretinal space, these samples were excluded from this analysis. Structurally defined bulbi (puncta) were counted manually in every fourth serial retinal cross-section containing graft. The number of hMITO^+^ bulbi was then summed and corrected (by division) to the total area of the corresponding graft. A bulbus was considered polarized when it was localized apically from the mCherry^+^ signal, at the notional level of the OLM, and was oriented toward the RPE. To assess the number of Müller glia, SOX2^+^ nuclei were quantified from 4–5 technical replicates of retinal cross-sections of areas with and without graft in each sample of each group. The values for each sample were averaged. To measure the ONL thickness, images were acquired in areas adjacent to the optic nerve in retinal cross-sections. Three measurements were obtained from 3–5 technical replicates in each sample of each group. The values for each sample were averaged. Given the apparent differences in the ONL thickness between the Cpfl1 and tg(Cpfl1/Rho^−/−^) retinae, blind assessments were not possible for the analyses of this study. Affinity Designer was used for the generation of the graphical abstract and schematic representation.

### Transmission electron microscopy

Mice were sacrificed, transplanted eyes were dissected to remove the muscle, cornea, lens, and optic nerve, and the rest was fixed with 4% formaldehyde in 100 mM phosphate buffer for 2 h at RT followed by a transfer to 1% formaldehyde/PBS for storage. The eyes were then further dissected under a fluorescence stereomicroscope (Leica MZ10F) to isolate labeled regions of interest (ROIs) in blocks of less than 1-mm size. These blocks were further processed for transmission electron microscopy according to a modified protocol developed for serial block-face scanning electron microscopy, which involves osmium tetroxide (OsO_4_), thiocarbohydrazide (TCH), and again OsO_4_ to generate enhanced membrane contrast.^2^^,^^31^^,^^33^ In brief, samples were post-fixed overnight in modified Karnovsky fixative (2% glutaraldehyde/2% formaldehyde in 100 mM PBS, pH 7.4), followed by washes in PBS and water and post fixation in a 2% aqueous OsO_4_ solution containing 1.5% potassium ferrocyanide and 2 mM CaCl_2_ (30 min on ice), washes in water, 1% TCH in water (20 min at RT), washes in water, and a second osmium contrasting step in 2% OsO_4_/water (30 min on ice). Samples were washed and en-bloc contrasted with 1% uranyl acetate/water for 2 h on ice, washed in water, and dehydrated in a graded series of ethanol/water mixtures (30%, 50%, 70%, 90%, and 96%, 20 min each), followed by three changes in pure water-free ethanol on a molecular sieve (30 min each). Samples were infiltrated into the epon substitute EMBed 812 (resin/ethanol mixtures: 1:3, 1:1, and 3:1 for 1 h each, followed by pure resin overnight and for 5 h) and finally cured at 65°C overnight. To identify ROIs in the blocks, semithin sections (1 μm) were prepared with a Leica UC6 ultramicrotome (Leica Microsystems, Wetzlar, Germany) using glass knives and stained with toluidine blue/borax for histological analysis. Once located, the ROIs were cut ultrathin (70 nm) using a diamond knife (Diatome, Nidau, Switzerland), after which the sections were collected on formvar-coated slot grids and stained with 2% aqueous uranyl acetate and with lead citrate. Mounted sections were analyzed with a JEM 1400Plus transmission electron microscope (JEOL, Freising, Germany) at 80 kV, and images and montages were taken with a Ruby digital camera (JEOL).

### Isolation and sample preparation of transplanted cells for single-cell transcriptomics

Retinae were dissected away from freshly enucleated eyes. The transplanted areas were isolated with a puncher tool under the RFP channel of a fluorescent microscope to detect the donor cells. Samples from three experimental eyes from each group were pooled together into one sequencing experiment to increase yield. The areas were dissociated first enzymatically with papain in a solution of 11 mM D-glucose in PBS, followed by mechanic trituration using a wide fire-polished glass pipette. The cells were carefully resuspended and visually inspected under a light microscope to determine cell concentration and quality. The single-cell suspensions were carefully mixed with the reverse transcription mix and were then loaded on the 10× Genomics (Prague, Czech Republic) Chromium system in a Chromium Single Cell G Chip (#PN-1000120) along with gel beads from Chromium Next GEM Single Cell 3′ v3.1 Gel Bead Kit v3.1 (#PN-1000122). Following the guidelines of the 10× Genomics Chromium Single Cell Kit v3.1 user manual (user guide ID: CG000315) and using the Chromium Next GEM Single Cell 3′ GEM Kit v3.1 (#PN-1000123), the droplets were directly subjected to reverse transcription, the emulsion was broken, and cDNA was purified using Dynabeads MyOne Silane (#PN-2000048). After amplification of cDNA with 11/12 cycles, 0.6× purification was performed with SPRIselect beads (Beckman Coulter, Krefeld, Germany, #B23319) to enrich cDNA fragments (>400 bp), and a quality control was performed on the fragment analyzer (using the DNF-473 NGS Fragment Kit/DND474 NGS High Sensitivity Kit, Agilent, Waldbronn, Germany). The 10× Genomics scRNA-seq library preparation—involving fragmentation, dA-tailing, adapter ligation, and a 11/13 cycles indexing PCR—was performed based on the manufacturer’s protocol using the 10× Genomics Library Construction Kit (#PN-1000190). Due to the abundance of cells in Cpfl1 recipients, the pooled samples were split into two technical replicates. Therefore, two libraries were created for Cpfl1 recipients and one for tg(Cpfl1/Rho^−/−^) recipients, which were sequenced on an Illumina Novaseq6000 in paired-end mode (R_1_/R_2_: 100 cycles; I_1_/I_2_: 10 cycles), generating ∼140–360 million fragment pairs.

### Transcriptome analysis

The raw sequencing data was processed with the count command of the Cell Ranger software (v.6.1.2) provided by 10× Genomics (https://www.10xgenomics.com). Since samples were a mix of human and mouse cells, Cell Ranger was run using a combined reference (GRCh38 and mm10, 2020-A, https://www.10xgenomics.com) to separate the 10× cell barcodes by species. Cell Ranger indices were built following the instructions provided by 10× Genomics (https://www.10xgenomics.com/support/software/cellranger/downloads/cr-ref-build-steps, 2020-A). Analysis of the 10× Genomics scRNA-seq data was performed on Seurat (v.5.3.0)^34^ in Rstudio (v.2025.09.0+387). Quality control was performed to filter for cells of poor quality by adjusting the parameters for percentage of mitochondrial genes (<15%) expressed and number of transcripts present (200–7,500). Doublet detection was not performed. Post quality control, the number of donor cells in Cpfl1 recipients reduced to 1,127 from 1,287, while in tg(Cpfl1/Rho^−/−^) recipients it reduced to 1,295 from 1,459. Data were then normalized using the method LogNormalize with a scale factor of 10,000, scaled, and integrated using the method CCAIntegration. The pan-photoreceptor markers *CRX* and *RCVRN* were used to identify the photoreceptor identity of donor cells. The rod fraction was annotated based on the expression of *PDE6A*, *NR2E3*, *RHO*, and *SAG*, while the cone fraction was annotated based on the expression of *GNAT2*, *ARR3*, and *OPN1MW*. Expression of selected genes was visualized using the DoHeatmap package (v.5.4.0) from Seurat. To assess the DEGs in the cone fractions of Cpfl1 and tg(Cpfl1/Rho^−/−^) recipients, the Wilcoxon rank sum statistical test was used. The top 30 DEGs, with an adjusted *p* value of <0.05 and a threshold fold change of log_2_(2), were denoted in the volcano plot using the package ggplot2 (v.4.0.3).

### Statistics

Statistical tests of histological samples were performed in GraphPad Prism (v.10.6.1). For the graft volume analysis as well as the hMITO^+^ puncta quantifications, the values passed the Kolmogorov-Smirnov normality test and were then analyzed by the unpaired *t* test with Welch’s correction. As the sample size for the SOX2^+^ nuclei and the ONL thickness analyses was not sufficient to assess normality, the non-parametric Wilcoxon matched-pairs signed-rank test and the Kruskal-Wallis test were used, respectively. Values are portrayed as mean (SD).

### Study approval

Animal experiments were approved by the ethics committee of the Dresden University of Technology and the State Directorate of Dresden (TVV 38/2019, TVV 4/2025). All regulations from the European Union, the Animal Welfare Act from Germany, and the statement on the Use of Animals in Ophthalmic and Vision Research from the Association for Research in Vision and Ophthalmology were strictly followed.

## Data and code availability

The data generated in this study are available via controlled access in the German Human Genome-Phenome Archive (GHGA, data.ghga.de) under GHGA: https://data.ghga.de/study/GHGAS28278350225897. Further details, including the data access policy for the study, can be found there.

## Acknowledgments

The authors would like to thank the staff of the Light Microscope, Flow Cytometry, Dresden-concept Genome Center, and Stem Cell Engineering and Animal facilities of the CRTD/CMCB for their assistance with AxioScan imaging, cell sorting, scRNA-seq, cell culturing, and mouse maintenance, respectively. Further thanks go to Sabine Klaußner, Jochen Hentschel, and Susanne Ferguson for technical assistance. This work was supported by grants from the Deutsche Forschungsgemeinschaft (DFG) AD 375/7-1 and AD 375/7-2 (within the SPP2127; to M.A.) and AD 375/9-1 (to M.A.), and the Foundation Fighting Blindness TA-RM-0522-0824-TUD-TRAP (to M.A.). The study was co-funded by the ERASMUS+ program of the European Union (2024-1-EL01-KA131-HED-000210895, to A.M.). Views and opinions expressed are, however, those of the authors only and do not necessarily reflect those of the European Union or the State Scholarships Foundation (IKY). Neither the European Union nor the granting authority can be held responsible for them.

## Author contributions

M.P. designed and performed experiments, analyzed and visualized data, and wrote the manuscript; K.T. participated in experiment design and data curation; T.K. performed electron microscopy imaging and contributed to the methodology part of the manuscript; J.H. performed OCT; A.M. and C.P. participated in data analysis; B.C.-S.M. performed cell-culture experiments and contributed to the methodology part of the manuscript; F.R. supervised the analysis of the RNA-seq data; and M.A. conceptualized the project and supervised, reviewed, and edited the manuscript.

## Declaration of interests

The authors declare no competing interests.

## Supplemental information

Document S1. Figures S1–S5 and Tables S1 and S2

Document S2. Article plus supplemental information
