# Supplementary material for: Transplanted human photoreceptors differentially survive, incorporate, and mature in mildly and severely degenerated mouse retinae
Source: Mol Ther Adv. 2026 Oct 14;34(4):201866. doi: 10.1016/j.omta.2026.201866 (PMC13738407; doi:10.1016/j.omta.2026.201866)
Supplement: Supplementary file 1 [file mmc1.pdf]

OMTA, Volume *34*

Supplemental information

Transplanted human photoreceptors differentially survive, incorporate, and mature in mildly and severely degenerated mouse retinae

Maria Pavlou, Karen Tessmer, Juliane Hammer, Thomas Kurth, Aikaterini Makri, Celine Palitza, Berta Coll-San Martin, Fabian Rost, and Marius Ader

SUPPLEMENTAL INFORMATION

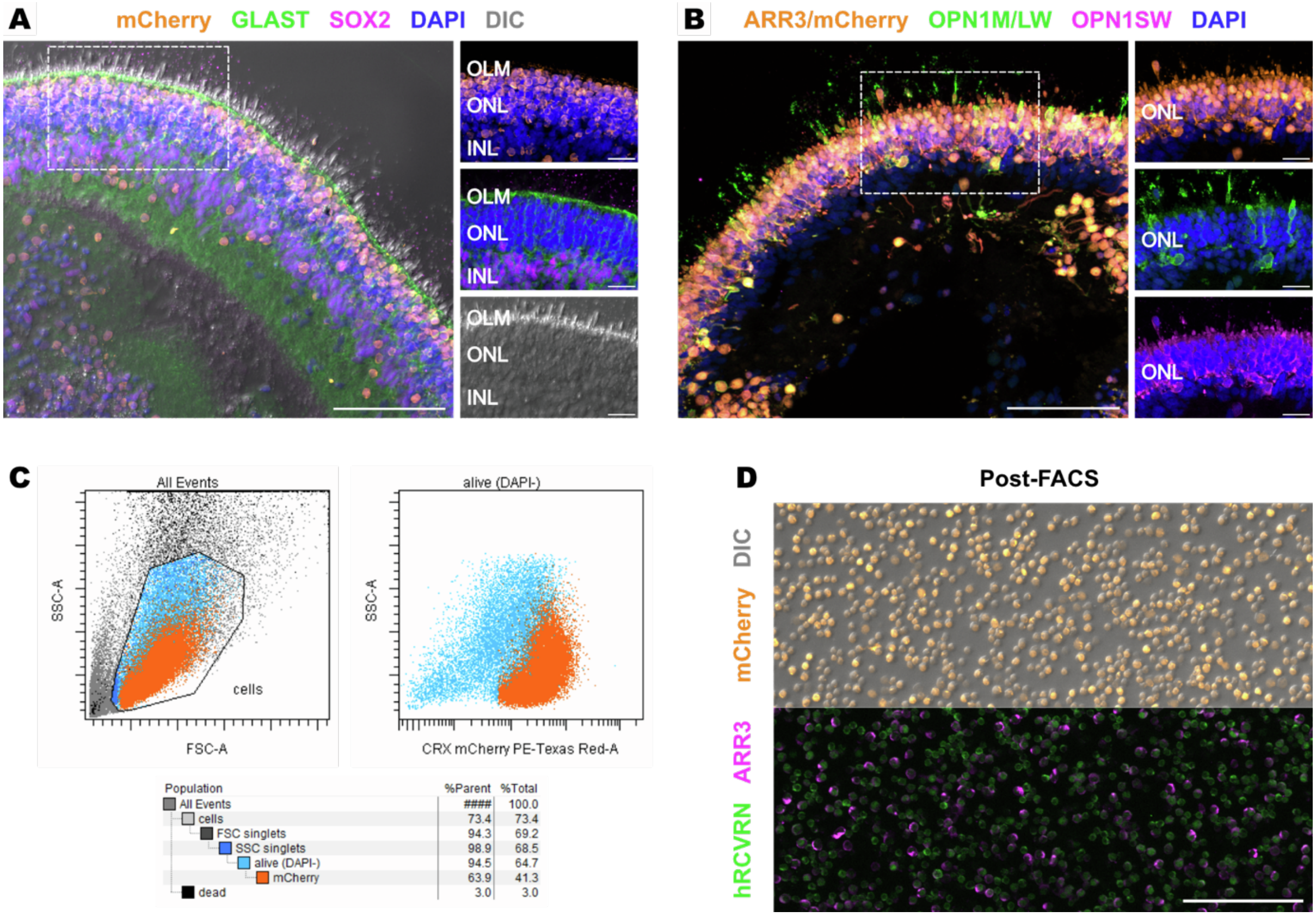

Population	%Parent	%Total
All Events	####	100.0
cells	73.4	73.4
FSC singlets	94.3	69.2
SSC singlets	98.9	68.5
alive (DAPI-)	94.5	64.7
mCherry	63.9	41.3
dead	3.0	3.0

Figure S1. D200 human retinal organoids generate transplantable photoreceptors. (A) D200 HROs present a laminated structure with Müller glia nuclei (SOX2, magenta) residing underneath photoreceptor nuclei (mCherry, orange), while their processes (GLAST, green) extend apically forming a notional OLM. (B) Photoreceptors present their distinctive morphology as seen with the cytosolic expression of cone arrestin (ARR3, orange), and express short wave-length sensitive opsins (OPN1SW, magenta) as well as medium/long-wavelength opsins (OPN1L/MW, green). (C) FACS allows the isolation and enrichment of mCherry$^+$ cells (orange) - gating indicates the target cell fraction. (D) Post-FACS mCherry$^+$ cells lose their distinctive photoreceptor morphology and round up with the majority expressing the pan-photoreceptor marker hRCVRN (green), and some expressing the cone-specific marker ARR3 (magenta). OLM: outer limiting membrane; ONL: outer nuclear layer; INL: inner nuclear layer; SOX2: SRY-box transcription factor 2; GLAST: glutamate/aspartate transporter 1; DIC: differential interference contrast; ARR3: arrestin 3; OPN1L/MW: opsin 1 long/medium wave sensitive;

OPN1SW: opsin 1 short wave sensitive; FACS: fluorescence-activated cell sorting; FSC: forward scatter; SSC: side scatter; hRCVRN: human-specific recoverin. Scale bars: 100 µm (Insets: 25 µm).

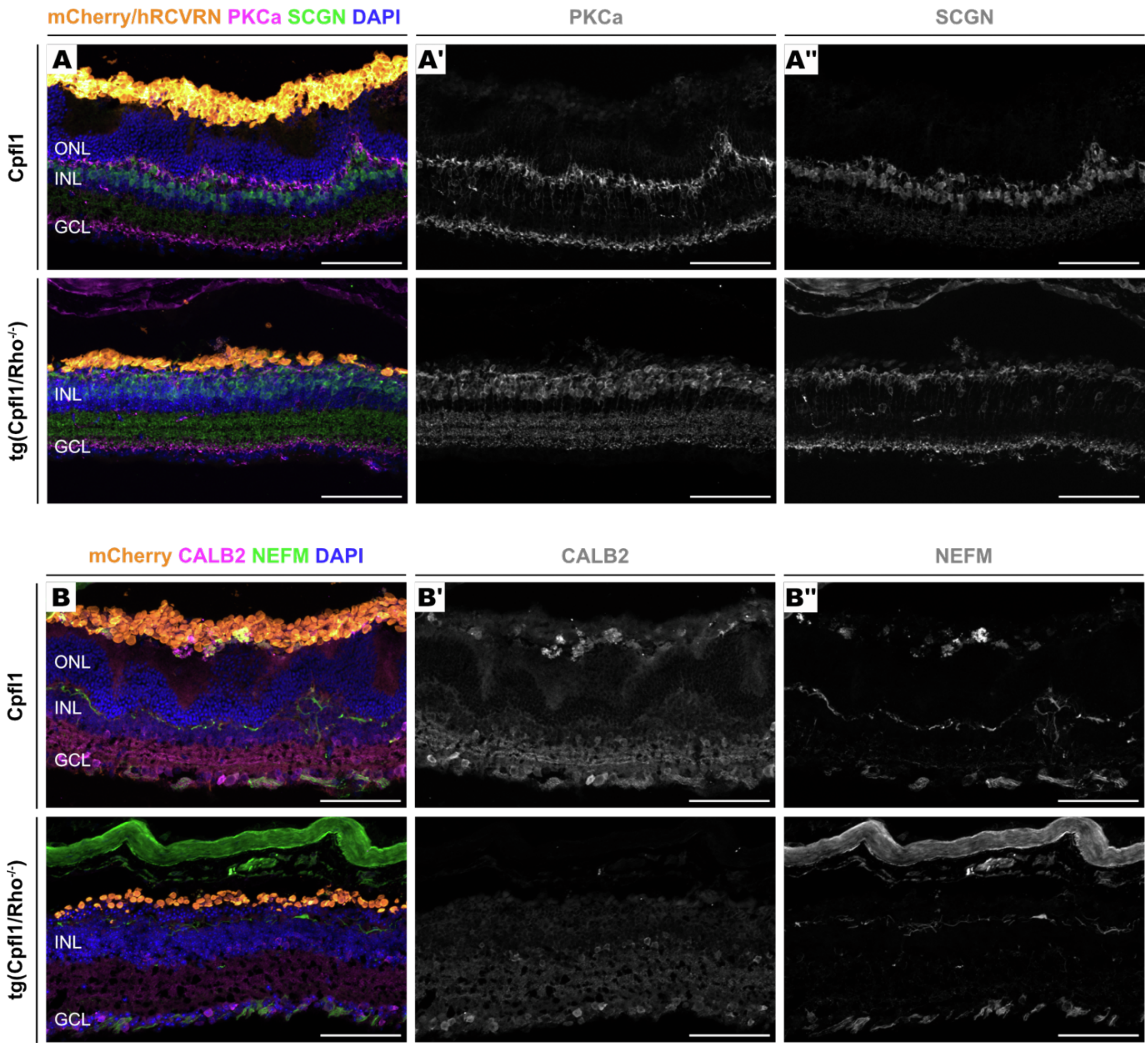

Figure S2. Bipolar, amacrine and horizontal cells do not interact with donor photoreceptors at 3 wpt. (A) Labelling against protein kinase C alpha (PKCa, magenta) and secretagogin (SCGN, green) shows no interactions between bipolar cells and donor photoreceptors (mCherry, orange; hRCVRN, green) at 3 wpt. (B) Labelling against calretinin (CALB2, magenta) and neurofilament medium chain (NEFM, green) shows no interactions between amacrine/horizontal cells, respectively, and donor photoreceptors (mCherry, orange) at 3 wpt. ONL: outer nuclear layer; INL: inner nuclear layer; GCL: ganglion cell layer; hRCVRN: human-specific recoverin (hRCVRN). Scale bar: 100 µm.

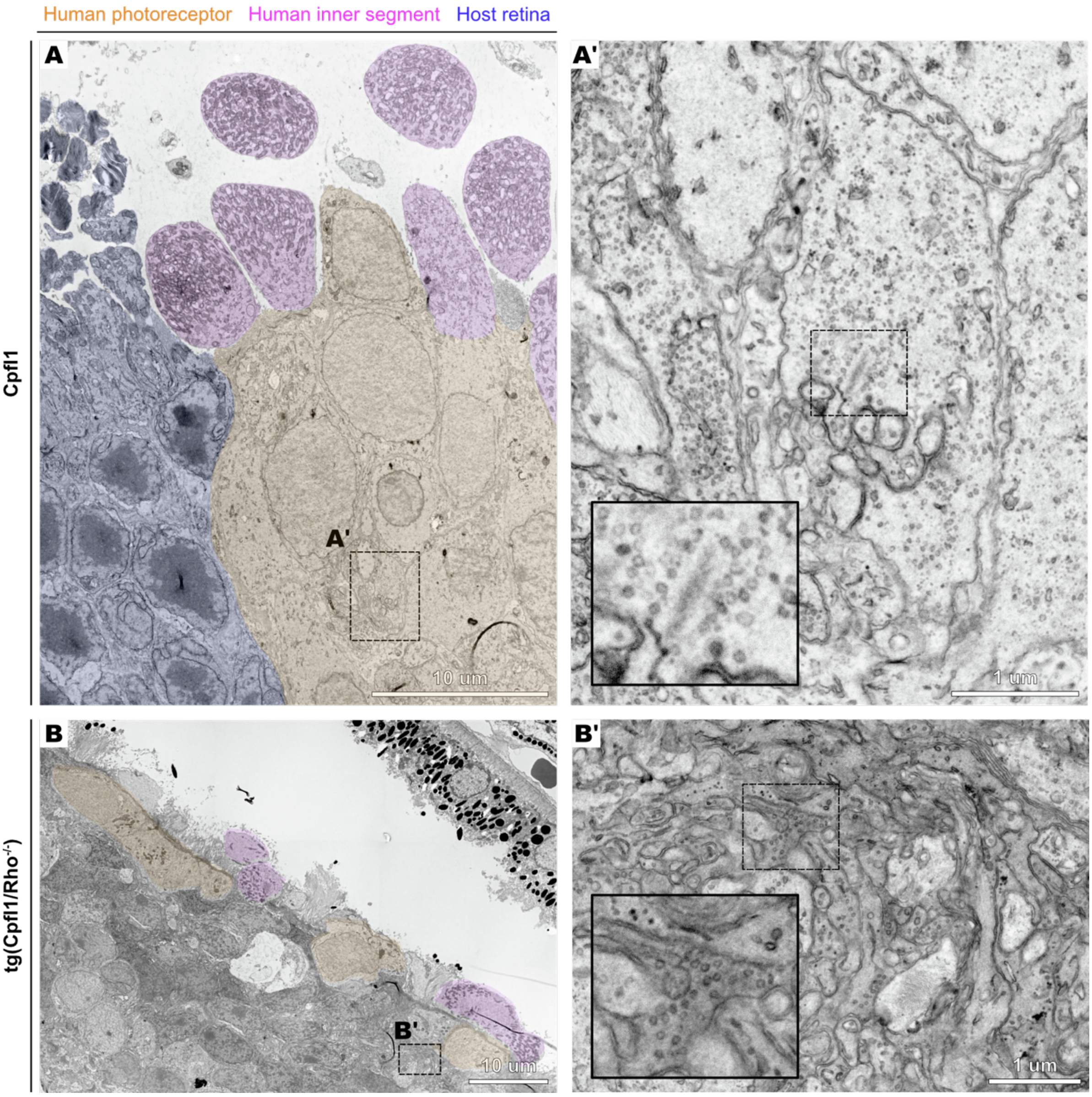

Figure S3. TEM reveals ribbon synapse formation by donor photoreceptors in mildly degenerated host. (A, A') Example of a mature synapse containing a ribbon and surrounding vesicles generated by an integrated donor photoreceptor at 26 wpt. (B - identical to Figure 5E, B') Very rarely premature synaptic structures are identified in tg(Cpfl1/Rho$^{-/-}$) hosts at 26 wpt.

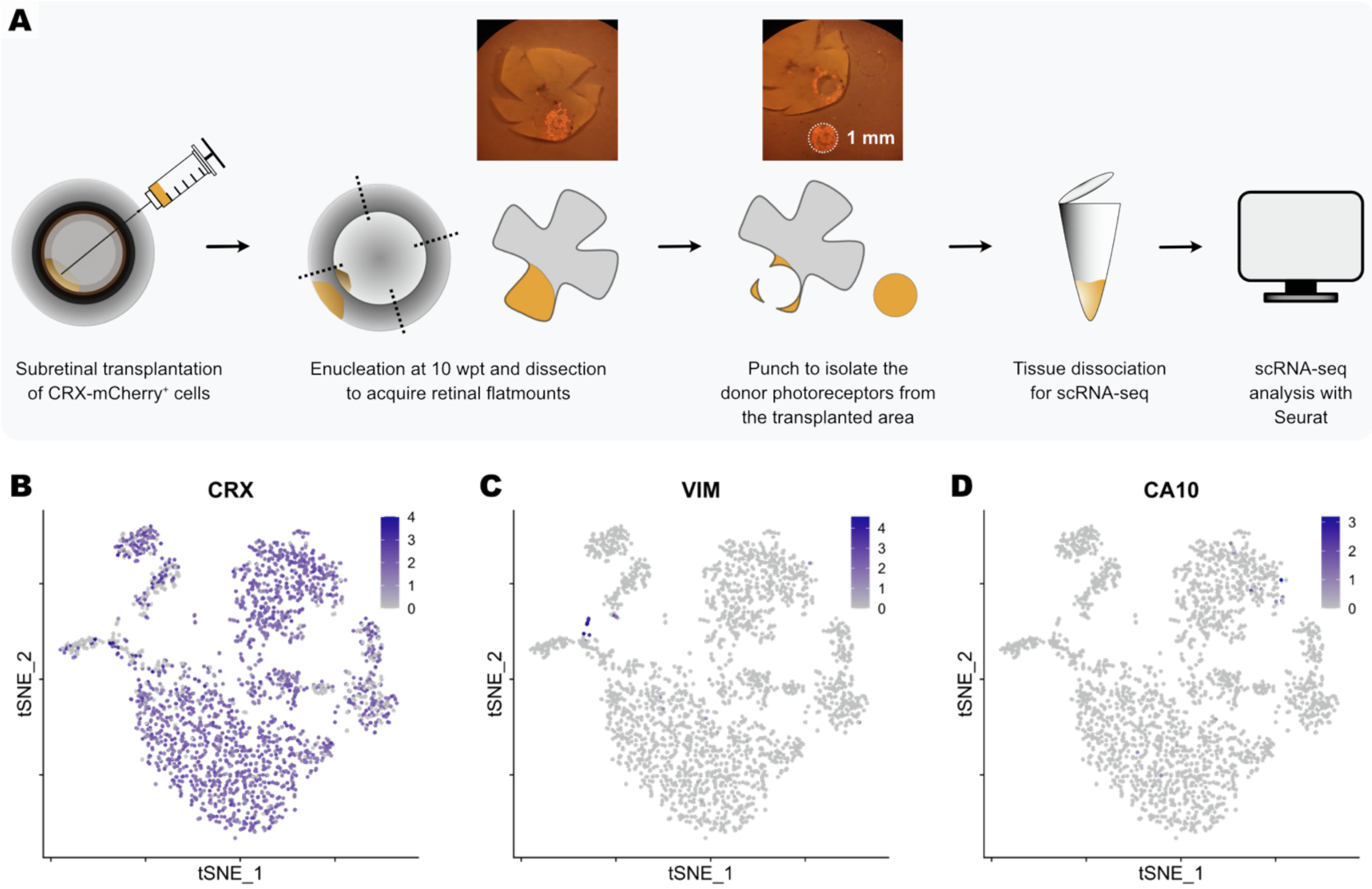

Figure S4. Evaluation of donor cell population through scRNA-seq analysis. (A) Pipeline of isolation of human transplanted cells for scRNA-seq. (B) t-SNE plot showing cone-rod homeobox (*CRX*) transcript expression in donor cell population. (C) t-SNE plot showing rare vimentin (*VIM*) transcript expression (specific for macroglia/Müller glia) in donor cell population. (D) t-SNE plot showing rare carbonic anhydrase 10 (*CA10*) transcript expression (specific for bipolar cells) in donor cell population.

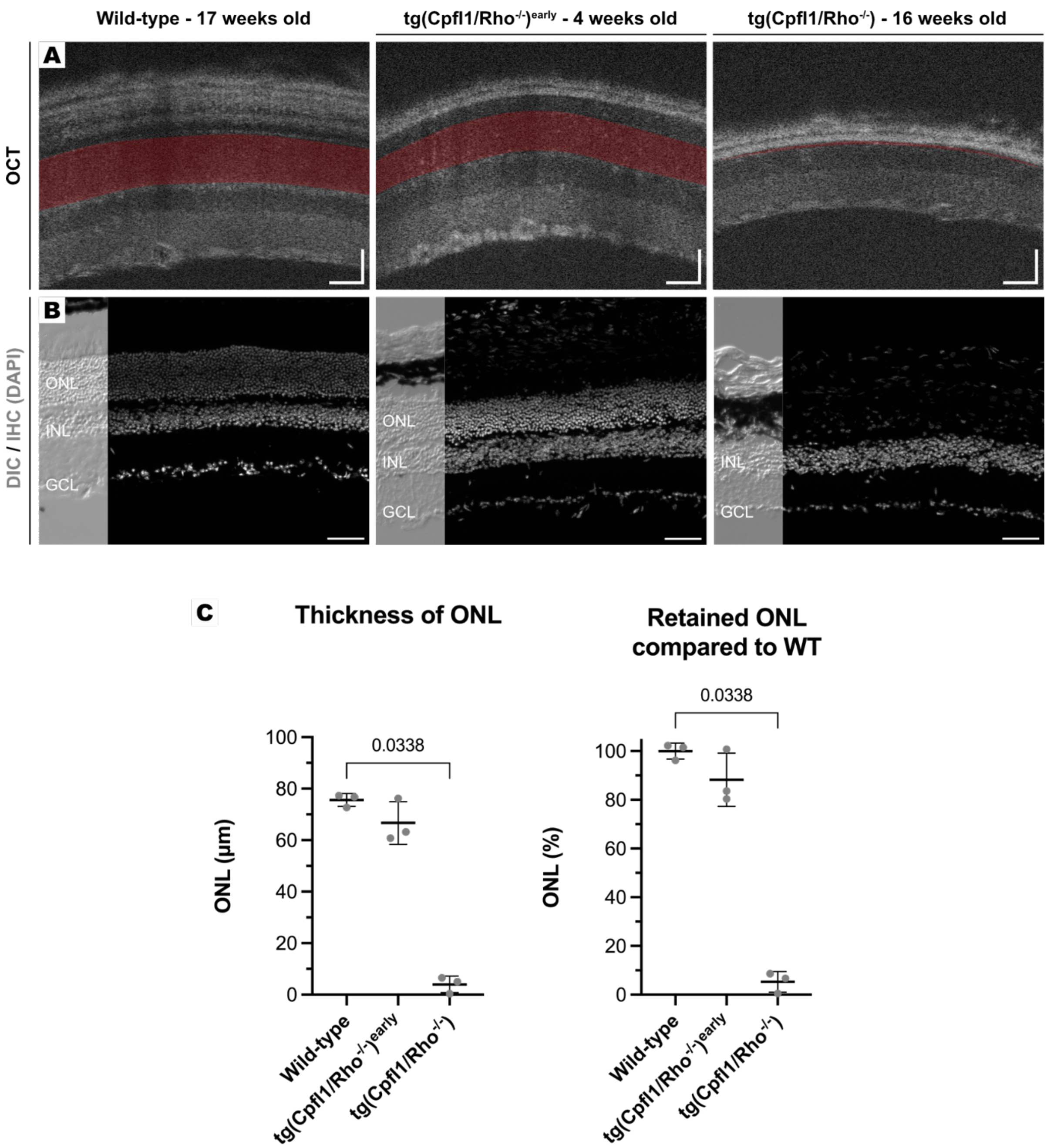

Figure S5. tg(Cpfl1/Rho$^{-/-}$) mice present age-dependent retinal degeneration. Progressive decrease of the ONL (marked in red) in tg(Cpfl1/Rho$^{-/-}$) mice at ages 4 and 16 weeks is visible by (A) *in vivo* optical coherence tomography (OCT) imaging, and (B) differential interference contrast (DIC) imaging and DAPI staining on cross-sections. Images were acquired from central retinal cross-sections areas adjacent to optic nerve. Scale bar: 50 µm. (C) Quantification of the ONL thickness in disease models. Each point represents an eye. n = 3 (N = 2) for WT, n = 3 (N = 3) for tg(Cpfl1/Rho$^{-/-}$)^{early}, and n = 3 (N = 3) for tg(Cpfl1/Rho$^{-/-}$) mice. Kruskal-Wallis test, line at mean (SD). ONL: outer nuclear layer; INL: inner nuclear layer; GCL: ganglion cell layer.

Table S1. Primary antibodies

Protein target	Abbreviation	Company / City, Country/ Catalogue number	Dilution
Arrestin 3	ARR3	Novus Biologicals / Wiesbaden Nordenstadt, Germany / NBP1-37003	1:100
Rhodopsin	RHO	Sigma-Aldrich / Taufkirchen, Germany / O4886	1:1000
Membrane protein palmitoylated 5	MPP5	Proteintech / Planegg-Martinsried, Germany / 17710-1-AP	1:200
Glial fibrillary acidic protein	GFAP	Sigma-Aldrich / Taufkirchen, Germany / 345860	1:500
Glutamine synthetase	GLUL	BD Biosciences / Heidelberg, Germany / 610517	1:250
mCherry	mCherry	Abcam / Darmstadt, Germany / ab205402	1:1000
Human-specific recoverin	hRCVRN	Sigma-Aldrich / Taufkirchen, Germany / ZRB1107	1:500
Recoverin	RCVRN	Sigma-Aldrich / Taufkirchen, Germany / AB5585	1: 1500
SRY-box transcription factor 2	SOX2	Santa Cruz Biotechnology / Heidelberg, Germany / sc-17320	1:150
Human-specific mitochondrial content	hMITO	Abcam / Darmstadt, Germany / ab92824	1:500
Opsin 1 medium/long wave sensitive	OPN1M/LW	Sigma-Aldrich / Taufkirchen, Germany / AB5405	1:1000
Opsin 1 short wave sensitive	OPN1SW	Santa Cruz Biotechnology / Heidelberg, Germany / sc-14363	1:1000
Glutamate/aspartate transporter	GLAST	Miltenyi Biotec / Bergisch Gladbach, Germany / 130-095-822	1:200
Protein kinase C alpha	PKCa	Santa Cruz Biotechnology / Heidelberg, Germany / sc-8393	1:500
Secretagogin	SCGN	BioVendor / Eching, Germany / RD184120100	1:400
Calretinin	CALB2	Swant / Kehrsatz, Switzerland / 7699/4	1:1000
Neurofilament medium chain	NEFM	DSHB / Iowa City (Iowa), USA / AB_531793	1:100

Table S2. Secondary antibodies

Product	Company / City, Country / Catalogue number
Cy™5 AffiniPure® Donkey Anti-Rabbit IgG (H+L)	Jackson ImmunoResearch Laboratories / Hamburg, Germany / 711-175-152
Cy™3 AffiniPure® Donkey Anti-Rabbit IgG (H+L)	Jackson ImmunoResearch Laboratories / Hamburg, Germany / 711-165-152
Alexa Fluor® 488 AffiniPure® Donkey Anti-Rabbit IgG (H+L)	Jackson ImmunoResearch Laboratories / Hamburg, Germany / 711-545-152
Cy™5 AffiniPure® Donkey Anti-Mouse IgG (H+L)	Jackson ImmunoResearch Laboratories / Hamburg, Germany / 715-175-150
Cy™3 AffiniPure® Goat Anti-Mouse IgG (H+L)	Jackson ImmunoResearch Laboratories / Hamburg, Germany / 115-165-146
Cy™2 AffiniPure™ Donkey Anti-Mouse IgG (H+L)	Jackson ImmunoResearch Laboratories / Hamburg, Germany / 715-225-150
Cy™3 AffiniPure® Donkey Anti-Chicken IgY (IgG) (H+L)	Jackson ImmunoResearch Laboratories / Hamburg, Germany / 703-165-155
Cy™5 AffiniPure® Donkey Anti-Goat IgG (H+L)	Jackson ImmunoResearch Laboratories / Hamburg, Germany / 705-175-147
Cy™3 AffiniPure® Donkey Anti-Goat IgG (H+L)	Jackson ImmunoResearch Laboratories / Hamburg, Germany / 705-165-147
Alexa Fluor® 488 AffiniPure® Donkey Anti-Goat IgG (H+L)	Jackson ImmunoResearch Laboratories / Hamburg, Germany / 705-545-147
Alexa Fluor® 488 AffiniPure® Donkey Anti-Sheep IgG (H+L)	Jackson ImmunoResearch Laboratories / Hamburg, Germany / 713-225-147
Alexa Fluor® 647 AffiniPure® Donkey Anti-Rat IgG (H+L)	Jackson ImmunoResearch Laboratories / Hamburg, Germany / 712-605-153
DAPI	Sigma-Aldrich / Taufkirchen, Germany / D9542
